# Targeting *Staphylococcus aureus* Biofilms Through Aurisin Derivatives From *Neonothopanus nambi* of African Origin

**DOI:** 10.1002/cbdv.202503741

**Published:** 2026-02-03

**Authors:** Syeda J. Khalid, Njogu M. Kimani, Yuanyuyue Huang, Mathias Müsken, Jan‐Peer Wennrich, Jonas Bentrup, Miroslav Kolařík, Josphat Clemet Matasyoh, Hedda Schrey

**Affiliations:** ^1^ Department of Microbial Drugs Helmholtz Centre for Infection Research GmbH (HZI) and German Centre for Infection Research (DZIF) Braunschweig Germany; ^2^ Institute of Microbiology Technische Universität Braunschweig Braunschweig Germany; ^3^ Institute For Organic and Analytical Chemistry University of Bremen Bremen Germany; ^4^ Department of Physical Sciences University of Embu Embu Kenya; ^5^ Central Facility for Microscopy Helmholtz Centre for Infection Research GmbH (HZI) and German Centre for Infection Research (DZIF) Braunschweig Germany; ^6^ Institute For Physical and Theoretical Chemistry University of Bremen Bremen Germany; ^7^ Institute of Microbiology Academy of Sciences of the Czech Republic Prague Czech Republic; ^8^ Department of Botany, Faculty of Science Charles University Prague Czech Republic; ^9^ Department of Chemistry Egerton University Njoro Kenya

**Keywords:** biofilm inhibition, fungal metabolites, natural products, *Neonothopanus nambi*, *Staphylococcus aureus*

## Abstract

Biofilm formation is a key survival strategy among microorganisms and a major factor contributing to chronic and treatment‐resistant infections. *Staphylococcus aureus* is a key biofilm‐forming pathogen associated with persistent and life‐threatening infections. As part of our ongoing search for novel anti‐biofilm agents from Basidiomycota of tropical rainforests, we investigated the metabolome of the African fungus *Neonothopanus nambi*. This study led to the isolation of four dimeric aristolane‐type sesquiterpenoids, aurisins D, B, A, and G (**1**–**4**), along with two monomeric sesquiterpenoids, nambinone C (**5**) and axinysone B (**6**), and methyl 4‐butyramidobenzoate (**7**). All compounds were assessed for their antimicrobial and cytotoxic activities, as well as their ability to inhibit and eradicate *S. aureus* biofilms. The dimeric sesquiterpenoids (**1**–**4**) exhibited potent antibiofilm effects, with aurisin B (**2**) inhibiting biofilm formation by over 70% at a concentration of 1 µg/mL, well below its minimum inhibitory concentration. Confocal laser scanning microscopy further confirmed the pronounced anti‐biofilm effects of aurisins D and B (**1** and **2**). Structure–activity relationship analysis suggests that both dimerization and hydroxylation contribute to enhanced activity. Despite some cytotoxic effects, these dimeric aristolane‐type sesquiterpenoids from *N. nambi* represent promising leads for the development of novel anti‐infective strategies targeting *S. aureus* biofilms, with potential applications beyond systemic use.

## Graphical abstract

This study describes the isolation and characterization of potent bioactive compounds from the fungal strain *Neonothopanus nambi*, followed by structural elucidation. The compounds shown are dimeric aristolane‐type sesquiterpenoids, identified as aurisins D (**1**) and B (**2**). Antibiofilm assays against *Staphylococcus aureus* demonstrate significant inhibition of biofilm formation by compounds **1** and **2** at concentrations of 2 µg/mL and 1 µg/mL, respectively. Confocal laser scanning microscopy provides visual evidence supporting the antibiofilm activity of these bioactive compounds.

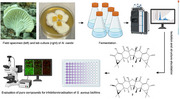

## 1 Introduction

Effective antimicrobial therapeutics are vital for infection treatment, yet antimicrobial resistance (AMR) has become a major global health threat. The 2022 GLASS report highlights a 15% rise in AMR rates since 2017, with 4.95 million deaths linked to resistant infections in 2019 [1, 2]. Biofilm formation further complicates treatment, as biofilms enhance microbial resistance to antibiotics and immune defenses, posing a significant challenge in managing chronic infections [3].

Biofilms are complex microbial communities encased in an extracellular polymeric substance (EPS) matrix, characterized by stable cell attachment and distinct phenotypic shifts. These communities, which may include bacteria, fungi, archaea, and protozoa, form intricate channel structures that regulate nutrient and gas exchange. Biofilms have a unique architecture that causes significant health and disease implications [4, 5]. These structured microbial consortia can colonize diverse biotic and abiotic surfaces, thriving in environments with adequate nutrients, humidity, and temperature [6]. Humans, with their rich biotic surfaces, are particularly susceptible to biofilm formation, often leading to infectious diseases [7].

As NIH reported in 2002, approximately 80% of microbial infections are biofilm‐related [8]. Among them, *Staphylococcus aureus* is a leading nosocomial pathogen known for its multidrug resistance. It is commonly associated with skin and soft tissue infections, and can cause severe conditions like endocarditis, pneumonia, and toxin‐mediated syndromes. *S. aureus* has developed resistance to multiple antibiotic classes, and the global rise of methicillin‐resistant (MRSA), vancomycin‐intermediate (VISA), and vancomycin‐resistant (VRSA) strains intensifies the threat [9, 10]. This escalating issue underscores the urgent need for novel antimicrobial agents to address multidrug‐resistant pathogens.

The discovery of penicillin marked a turning point in medicine, highlighting fungi as a rich source of bioactive compounds [11]. Since then, fungi have proven to be a valuable reservoir for novel pharmaceutical agents, with landmark discoveries like lefamulin, fingolimod, pleuromutilins, and enfumafungin, the latter leading to the development of ibrexafungerp. Lately, the derivatives of pleuromutilin, tiamulin, and retapamulin have gained approval for veterinary and human medical applications [12]. With around 40 000 species globally, Basidiomycota ranks as the second‐largest fungal phylum and a prolific source of bioactive metabolites [13]. Kavanagh et al. initially identified pleuromutilin, one of the first antibiotics derived from Basidiomycota [14]. Recent isolations of bioactive secondary metabolites from Basidiomycota have revealed a wide array of compounds, including monoterpenoids, sesquiterpenoids, diterpenoids, triterpenoids, tetraterpenoids, polyketides, and alkaloids. This diversity not only highlights the rich chemical repertoire of Basidiomycota but also underscores the correlation between chemical and biological diversity within this group [15].

Based on our screening for antimicrobial metabolites in general and antibiofilm compounds in particular in the African Basidiomycota collection from Arabuko Sokoke National Park, Kenya, *Neonothopanus nambi* was selected for further investigation due to the significant activity of its crude extract against *S. aureus* biofilms. *N. nambi*, a lesser‐known fungal species, has emerged as a potential treasure trove for secondary metabolites with significant pharmacological and industrial applications. Previous studies have demonstrated that *N. nambi* produces various bioactive secondary metabolites with notable activities, including antibacterial, antimalarial, antimycobacterial, cytotoxic, and anti‐nematode properties [16, 17, 18, 19].

Our investigation of *N. nambi* from Arabuko Sokoke National Park led to the isolation of seven compounds, including three dimeric sesquiterpenoids (**1**‐**3**) that were formerly patented in 2004 as derivatives of aurisin A (**4**) and described for their potential in controlling phytopathogenic microorganisms in agriculture [20]. In addition, we identified three previously reported compounds, nambinone C (**5**), axinysone B (**6**), and methyl 4‐butyramidobenzoate (**7**). In this study, we report their isolation and structural characterization, including the determination of the stereochemistry of aurisins D and B (**1** and **2**), along with their potent inhibitory and biofilm‐eradicating properties of aristolane‐type dimeric sesquiterpenoids against *S. aureus* biofilms.

## 2 Results and Discussion

### 2.1 Isolation and Structure Elucidation of Compounds **1**–**3**

*N. nambi* (CCC 1560) was isolated from its specimen collected in Arabuko Sokoke Forest, located in the coastal region of Kenya, characterized by a tropical climate and diverse vegetation.

The production of secondary metabolites was evaluated through cultivation in a range of liquid media (YM 6.3, ZM ½, Q6 ½, SNL, SYM, GDYP, and CM) and solid rice medium. Crude extracts were evaluated for their biofilm eradication potential against *S. aureus*, where supernatant and mycelial extracts from YM 6.3 medium achieved eradication of ca. 40% at 62.5 µg/mL of 24 h old biofilm. Metabolomic analysis of crude extracts, conducted using high‐resolution electrospray ionization mass spectrometry (HR‐ESIMS), showed the most distinct and promising chromatographic peaks in YM 6.3 medium. Even though similar metabolites were found in SNL and GDYP to a lesser extent, YM 6.3 was selected for upscale fermentation due to its better metabolic clarity. After scaled‐up fermentation of *N. nambi* in YM 6.3 medium (4.8 L), targeted isolation using size‐exclusion chromatography (SEC) followed by high‐performance liquid chromatography (HPLC) afforded the purification of compounds **1**–**7**. Among these, compounds **1**–**3** were obtained as the major constituents of the crude extract, while compounds **4**–**7** were present only in minor quantities.

**FIGURE 1:**
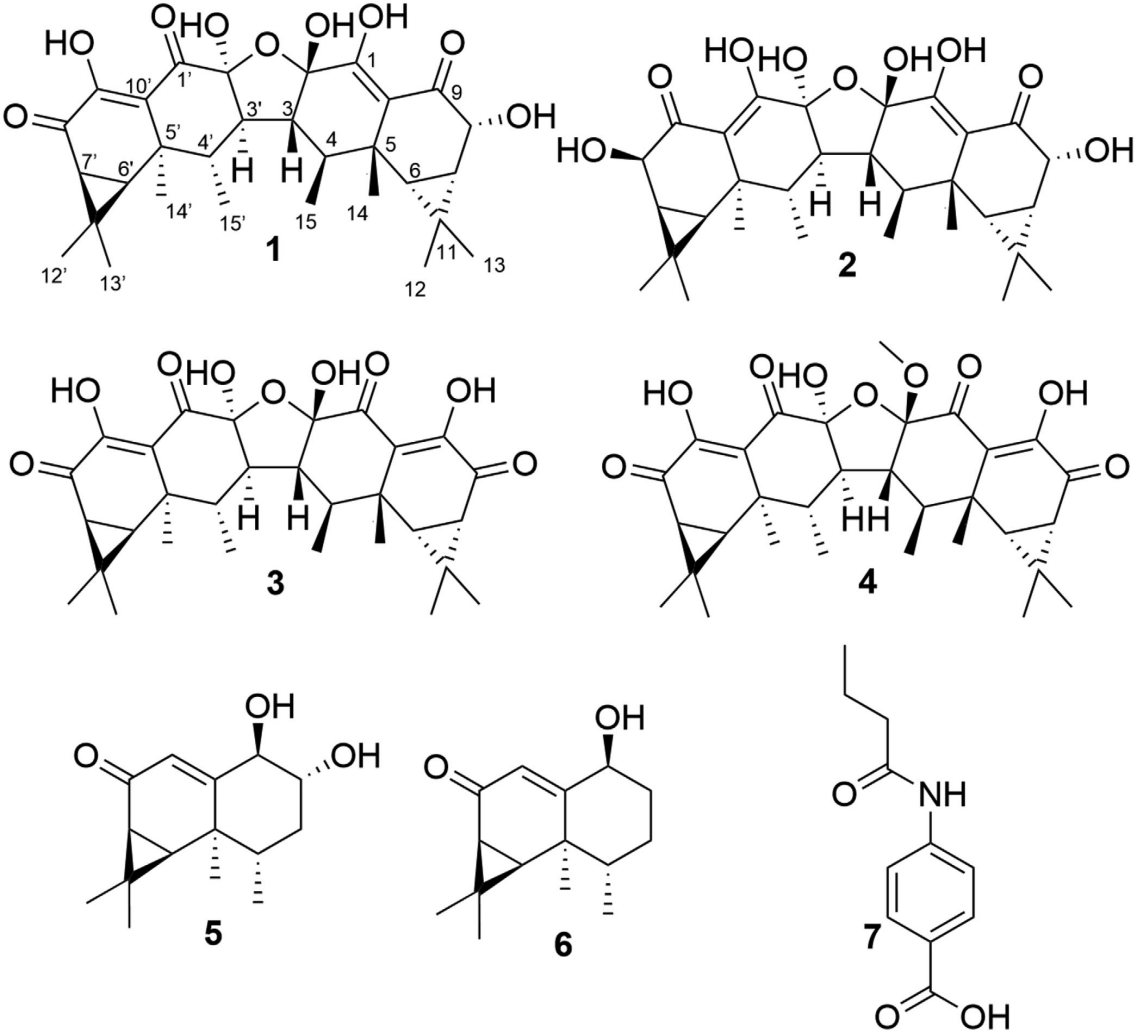
Chemical structures of aurisins D (**1**), B (**2**), A (**3**), G (**4**), together with nambinone C (**5**), axinysone B (**6**), and methyl 4‐butyramidobenzoate (**7**).

**FIGURE 2:**
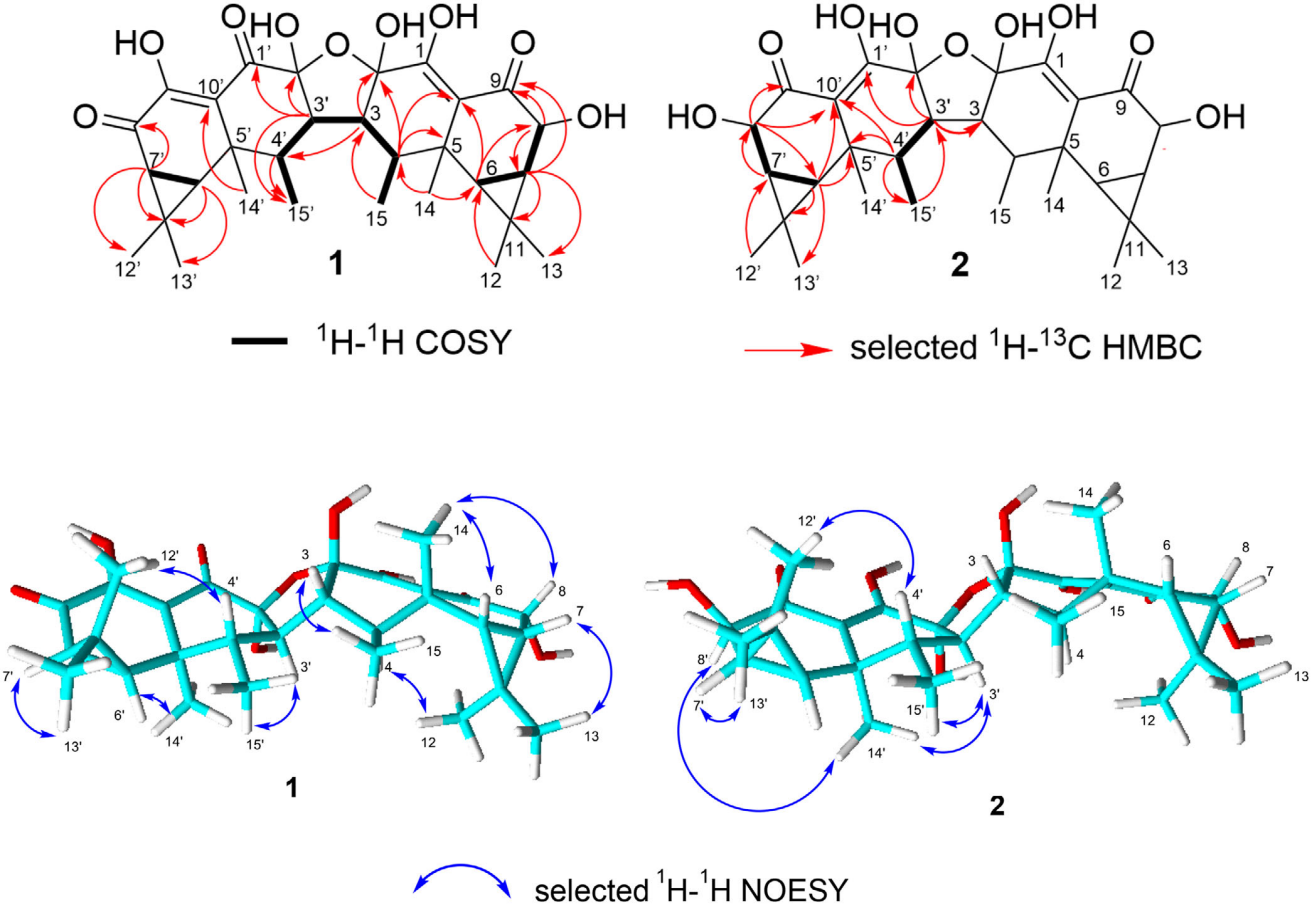
Key correlations of aurisins D (**1**) and B (**2**).

Compound **1** was obtained as a light‐yellow powder. The HR‐ESIMS spectrum showed a [M + H] ^+^ ion at *m/z* 543.2590 (calc. 543.2594 for C_30_H_39_O_9_^+^), corresponding to molecular formula C_30_H_38_O_9_ and indicating the presence of 12 degrees of unsaturation. The infrared (IR) spectrum confirmed the presence of hydroxyl groups (3449.70 cm^−^^1^) and conjugated ketone group (1677.19 cm^−^^1^). Additionally, a C‐H stretch was observed at 2973.13 cm^−1^. Analysis of ^1^H and heteronuclear single quantum coherence (HSQC) spectra indicated the presence of eight methyl groups (*δ*_C_ 13.4, 14.1, 15.7, 15.7, 24.7, 25.9, 29.7, and 30.6 ppm), and nine methine groups (*δ*_C_ 26.7, 32.6, 37.9, 37.9, 38.4, 40.0, 53.0, 53.8, and 69.8 ppm) (Table 1). In addition, 13 quaternary carbons were observed from the analysis of ^13^C nuclear magnetic resonance (NMR) (Table 1). Comparison of 1D/2D NMR data with those of aurisin A (**3**) showed a high degree of structural similarity [17], with a key distinction at C‐8. While aurisin A (**3**) contains a carbonyl group at C‐8, compound **1** features a hydroxyl group (*δ*_H_ 4.63 ppm, *δ*_C_ 69.8 ppm) at this position. A literature review revealed that a compound corresponding to **1** had previously been described in a patent by Boehlendorf et al. [20], under the name aurisin D, classified as a dimeric aristolane derivative. However, the patent lacked stereochemical analysis and a complete 1D/2D NMR data set. To address this, the relative configuration of compound **1** was elucidated through key nuclear Overhauser effect spectroscopy (NOESY) correlations. The NOESY spectrum exhibited cross peaks between H‐3 and H_3_‐15, H‐3’ and H_3_‐15’, H‐4 and H_3_‐12, H‐4’ and H_3_‐12’, H‐6’ and H_3_‐14’, H‐7 and H_3_‐13, H‐7’ and H_3_‐13’, as well as between H_3_‐14 and H‐6/H‐8 (Figure 2), indicating the orientation of those protons in **1**. Furthermore, the measured electronic circular dichroism (ECD) spectrum of **1** closely resembled that of aurisin A (**3**) (see Figure S35), whose absolute configuration has been established via X‐ray crystallography. This similarity indicates that aurisin D (**1**) possesses the same absolute configuration as **3**, as depicted in Figure 1.

**TABLE 1:** ^1^H (500 MHz) and ^13^C nuclear magnetic resonance (NMR) (125 MHz) data for **1** (CD_3_CN), **2** (CDCl_3_) at 298 K.

	1		2	
No.	*δ*_C_, type	*δ*_H_ (*J* in Hz)	*δ*_C_, type	*δ*_H_ (*J* in Hz)
1	174.2, C		171.2, C	
2	101.9, C		101.4, C	
3	53.8, CH	2.35, m	51.8, CH	2.37–2.27^*^
4	40.0, CH	2.26, dq (12.7, 6.3)	39.2, CH	2.37–2.27^*^
5	38.6, C		38.0, C	
6	32.6, CH	1.11, d (9.8)	32.0, CH	1.07^*^
7	26.7, CH	1.40, dd (9.8, 6.2)	26.8, CH	1.50, dd (9.7, 6.2)
8	69.8, CH	4.63, d (6.2)	69.7, CH	4.62, d (6.2)
9	202.9, C		202.0, C	
10	112.1, C		111.0, C	
11	20.3, C		20.2, C	
12	15.7, CH_3_	1.02, s	15.4, CH_3_	1.06, s
13	30.6, CH_3_	1.09, s	30.6, CH_3_	1.11, s
14	24.7, CH_3_	1.19, s	24.8, CH_3_	1.19, s
15	13.4, CH_3_	1.15, d (6.4)	13.4 CH_3_	1.15, d (6.8)
1'	201.3, C		171.2, C	
2'	101.6, C		101.4, C	
3'	53.0, CH	2.35, m	51.8, CH	2.37–2.27^*^
4'	37.9, CH	2.47, m	39.2, CH	2.37–2.27^*^
5'	38.1, C		38.0, C	
6'	38.4, CH	1.64, d (7.9)	32.0, CH	1.07^*^
7'	37.9, CH	1.98, d (7.9)	26.8, CH	1.50, dd (9.7, 6.2)
8'	191.8, C		69.7, CH	4.62, d (6.2)
9'	161.7, C		202.0 C	
10'	120.2, C		111.0, C	
11'	28.7, C		20.2, C	
12'	15.7, CH_3_	1.25, s	15.4, CH_3_	1.06, s
13'	29.7, CH_3_	1.27, s	30.6, CH_3_	1.11, s
14'	25.9, CH_3_	1.17, s	24.8, CH_3_	1.19, s
15'	14.1, CH_3_	1.22, d (6.7)	13.4, CH_3_	1.15, d (6.8)
OH				9‐OH;14.88, s 2‐OH; 3.74, s

* Overlapping signals.

Compound **2** was obtained as a white powder. The HR‐ESIMS spectrum showed a [M + H] ^+^ ion at *m/z* 545.2745 (calc. 545.2745 for C_30_H_41_O_9_^+^), corresponding to the molecular formula C_30_H_40_O_9_ and indicating 11 degrees of unsaturation. The IR spectrum confirmed the presence of hydroxyl groups (3449.70 cm^−^^1^) and conjugated ketone group (1677.19 cm^−^^1^). Analysis of the NMR spectra, in conjunction with MS data, suggested that compound **2** is a symmetrical dimer. This conclusion is supported by the observation of only half the expected proton and carbon signals in the spectra (S21, S22), the similarity of the *δ* values (Table 1), and the presence of a cross‐peak between H‐3’ (*δ*_H_ 2.37 – 2.27 ppm) and C‐3 (*δ*_C_ 51.8 ppm) within the heteronuclear multiple bond correlation spectrum. For the monomeric unit, ^1^H, HSQC, and ^13^C spectra analysis revealed the presence of four methyl groups (*δ*_C_ 13.4, 15.4, 24.8, and 30.6 ppm), five methine groups (*δ*_C_ 26.8, 32.0, 39.2, 51.8, and 69.7 ppm) and six quaternary carbons (*δ*_C_ 20.2, 38.0, 101.4, 111.0, 171.2, and 202.0 ppm; Table 1).  Compared to aurisin D (**1**), the 1D/2D NMR spectra of compound **2** showed an additional hydroxyl group at C‐8’ (*δ*_C_ 69.7 ppm), supported by a correlated spectroscopy correlation between H‐7’/H‐8’. A literature review revealed that this compound matched the structure of aurisin B, previously described in the same patent by Boehlendorf et al. [20], though the patent also lacked stereochemical data and a comprehensive NMR analysis. Therefore, the relative configuration of **2** was determined via NOESY correlations and by comparison with those of aurisin D (**1**). The NOESY spectrum exhibited correlations between H‐3’ and H‐14’; H‐3’ and H‐15’; H‐4’ and H‐12’; H‐6’ and H‐15’; H‐7’ and H‐13’ as well as between H‐8’ and H‐14’ (Figure 2), suggesting that compound **2** possesses a similar configuration to that of **1**. Furthermore, the ECD spectrum of aurisin B (**2**) closely resembles those of aurisins D and A (**1** and **3**), confirming that they share the same absolute configuration (Figure 1).

In addition to compounds **1** and **2**, two further known aurisin‐type sesquiterpenoids, aurisins A and G (**3** and **4**), were identified. Their structures were elucidated based on comprehensive HR‐ESI‐MS and 1D/2D NMR spectroscopic data, supported by comparison with literature [17, 20, 21]. Aurisin derivatives represent a rare class of dimeric aristolane sesquiterpenoids exhibiting diverse oxidation patterns and substitution motifs. Aurisin‐type sesquiterpenoids are previously reported from *Neonothopanus* [17, 22] and *Anthracophyllum* [21] species, as well as from two *Panus* strains described in the patent by Boehlendorf et al. [20]. The consistent occurrence of these metabolites in *Anthracophyllum* and *Neonothopanus* reflects their taxonomic placement in the family Omphalotaceae [23], indicating a likely conserved biosynthetic potential among its members. In contrast, the reported occurrence of aurisins in *Panus* (order: Polyporales; family: Panaceae) suggests a possible taxonomic misidentification of the source material, as a conserved biosynthetic pathway across such evolutionarily distant orders would be unusual.

Furthermore, two monomeric sesquiterpenoids with aristolane skeleton, nambinone C (**5**) and axinysone B (**6**), potentially biosynthetic precursors of aurisin‐type sesquiterpenoids, as well as a benzoate derivative, methyl 4‐butyramidobenzoate (**7**), were isolated [17, 22, 24].

## 3 Biological Evaluation

The antimicrobial and cytotoxic properties of compounds **1**–**7** were evaluated against a panel of bacterial and fungal strains, as well as selected mammalian and cancer cell lines. The antimicrobial screening included both clinically relevant pathogens and representative indicator strains. Overall, aurisins D, B, A, and G (**1**–**4**) exhibited the most pronounced bioactivity in antibacterial and cytotoxicity assays. Compounds **5**–**7** showed weak to no activity in all assays (Table 2).

**TABLE 2:** Cytotoxicity (IC_50_) and antimicrobial activity (MIC) of **1**–**7**.

Tested organisms/Cells	1	2	3	4	5	6	7	Epothilone B
**IC_50_ against mammalian cell lines (µg/mL)**								
Human endocervical adenocarcinoma (KB‐3.1)	0.51	0.33	0.25	0.27	n.a.	n.a.	n.a.	0.000058
Mouse fibroblast (L‐929)	22.0	7	22	6.8	n.i.	n.i.	n.i.	0.00090
Human epidermoid carcinoma (A‐431)	n.a.	n.a.	n.a.	n.d.	n.a.	n.a.	n.a.	0.00023
Human breast adenocarcinoma (MCF‐7)	0.13	0.1	0.17	0.27	n.i.	n.i.	23.0	0.00014
Human lung carcinoma (A‐549)	1.8	1.3	1.8	1.5	n.a.	n.a.	n.a.	0.000027
Human prostate carcinoma (PC‐3)	0.6	0.72	1.3	n.d.	n.a.	n.a.	n.a.	0.00012
**MIC against bacteria (µg/mL)**								**Ref**.
*Bacillus subtilis* (DSM 10)	2.0	2.0	4.1	n.d.	n.i.	n.i.	n.i.	16.6^O^
*Staphylococcus aureus* (DSM 346)	16.6	8.3	8.3	8.3	n.i.	n.i.	n.i.	0.42^G^

IC_50_ and MIC (µg/mL), n.a.: no activity, n.i.: no inhibition up to 37 µg/mL for IC_50_ and 66.6 µg/mL for MIC, n.d.: not determined, G: gentamycin, O: oxytetracycline.

The aristolane‐type dimeric sesquiterpenoids (**1**‐**4**) exhibited pronounced activity in the minimum inhibitory concentration (MIC) assay against *S. aureus* (compounds: **1**:16.6 µg/mL, **2**, **3**, and **4**: 8.3 µg/mL) and *B. subtilis* (compounds **1** and **2**: 2 µg/mL and **3**: 4 µg/mL), indicating specificity of activity against Gram‐positive bacteria (Table S4). Compounds **1**–**4** also exhibited pronounced cytotoxicity against the tested cell lines. However, aristolane‐type monomeric sesquiterpenoids, nambinone C (**5**), axinysone B (**6**), and methyl 4‐butyramidobenzoate (**7**), were not active against any of the tested microorganisms, nor did they show cytotoxicity against the tested cell lines.

**FIGURE 3:**
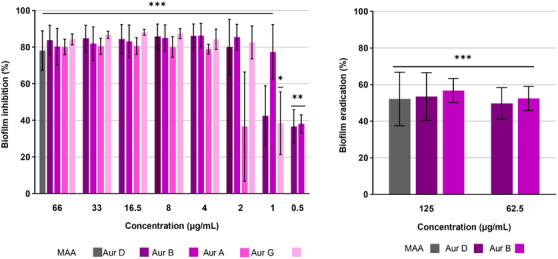
Assessment of biofilm inhibition/eradication of aurisins D, B, A, and G (**1**–**4**) via CV staining assay. Microporenic acid A (MAA) was used as a positive control. Methanol was used as a solvent control and taken as 100%. Error bars indicate standard deviation (SD) of data from three biological replicates, each with two independent technical repeats. Statistical significance was determined using one‐way analysis of variance (ANOVA), followed by Dunnett's post‐hoc test to compare each treatment group with the control. * *p* < 0.05, ** *p* < 0.01, and *** *p* < 0.001.

Compounds **1**–**7** were subsequently assessed for their ability to inhibit biofilm formation or eradicate preformed biofilms of *S. aureus* (Figure 3). Consistent with the trends observed in MIC and cytotoxicity assay, only the dimeric sesquiterpenoids (**1**–**4**) exhibited significant activity, whereas the monomeric analogues (**5** and **6**) remained inactive. Among the active compounds, the three aurisin derivatives D (**1**), B (**2**), and G (**4**) exhibited strong biofilm inhibitory effects, surpassing the efficacy of previously described compound aurisin A (**3**). Compound **1** effectively inhibited 73% of biofilm formation at a concentration of 2 µg/mL, while compound **2** achieved a comparable level of inhibition at just 1 µg/mL. Notably, both compounds exhibited approximately 40% biofilm inhibition even at a concentration as low as 0.5 µg/mL. Compound **4** retained significant activity up to a concentration of 2 µg/mL and was found to be more active than aurisin A (**3**), where significant activity lasted up to 4 µg/mL. These findings align with previous reports describing aurisin A (**3**) as a broad‐spectrum bioactive compound, including activity against *S. aureus* [18, 19, 25].

Importantly, all dimeric sesquiterpenoids (**1**–**4**) exhibited biofilm‐inhibitory activity at sub‐MIC concentrations, as compounds **1** and **2** exhibited, for example, anti‐biofilm activity at up to a three‐fold dilution [(MIC: 16 µg/mL, anti‐biofilm activity: 2 µg/mL for compound **1**), (MIC: 8.3 µg/mL, anti‐biofilm activity: 1 µg/mL for compound **2**)].

Confocal laser scanning microscopy (CLSM) imaging, together with analysis of red biovolume increase in comparison to total biovolume of 24 h established *S. aureus* biofilms, provided visual confirmation of the effect of tested compounds (**1** and **2**) on biofilm architecture.

**FIGURE 4:**
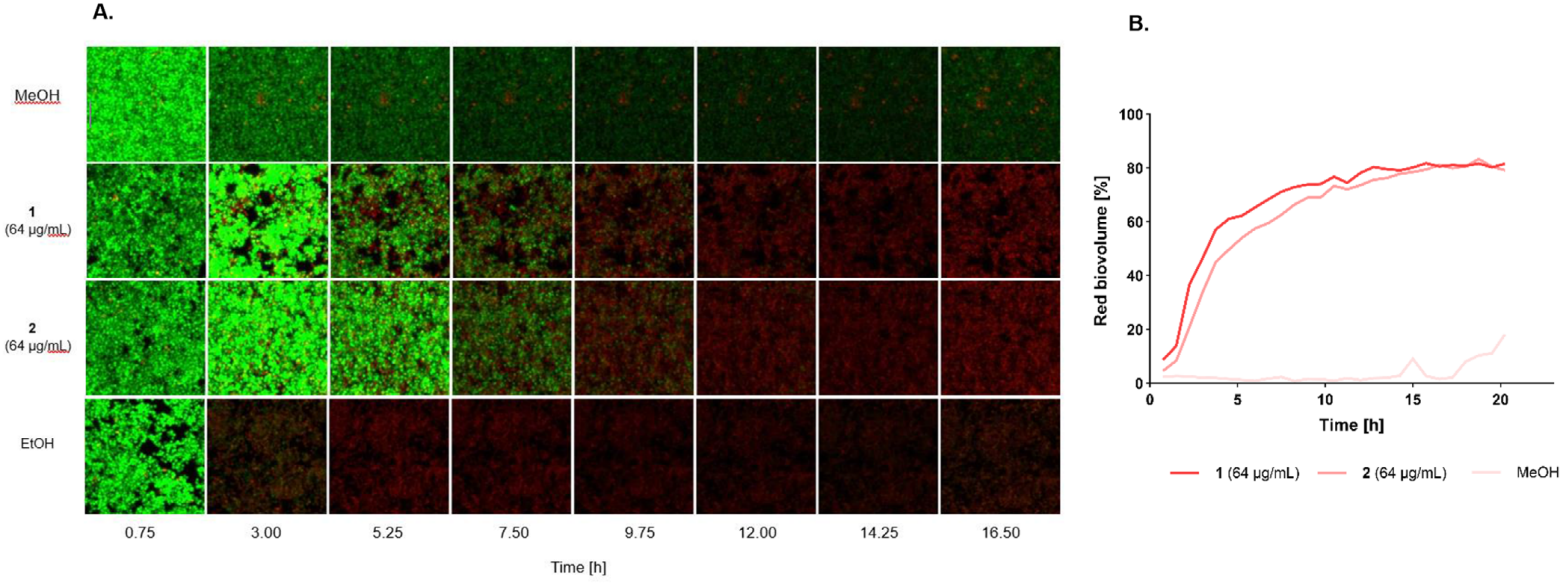
(A) Drug exposure of aurisis D (**1**) and B (**2**) on established, 24 h old biofilms captured using confocal laser scanning microscopy (CLSM). Biofilm viability was assessed using the BacLight Live/Dead Kit, showing live cells (green) and dead cells (red). Single images have a size of 37 × 37 µm. (B) Time‐lapse analysis of the increase in dead biovolume in 24 h established *S. aureus* biofilms, induced by aurisins D (**1**) and B (**2**). Methanol (MeOH) was used as a solvent control.

Where aurisin B (**2**) showed superior efficacy in CV biofilm inhibition and eradication assay (Figure 3), time‐lapse imaging revealed a slightly accelerated increase in dead biovolume for aurisin D (**1**) compared to aurisin B (**2**), starting 3 h after treatment (Figure 4). This observation suggests that aurisin D (**1**) more rapidly compromises cell viability, whereas aurisin B (**2**) exerts a more sustained inhibitory and eradication effect at lower concentrations, as seen through the CV biofilm inhibition assay.

Structural analysis of the aurisins (**1**–**4**) suggested that hydroxylation plays a crucial role in enhancing anti‐biofilm activity. Compound **1** contains one additional hydroxyl group at C‐1 compared to aurisin A (**3**), correlating with increased potency, while compound **2**, featuring two additional hydroxyl groups at C‐1 and C‐1’, retains efficacy at an even higher dilution. Aurisin G (**4**) differs from compound **3** by a methoxy group at C‐2 instead of a hydroxyl group, while retaining significant bioactivity, suggesting that although hydroxylation generally enhances potency, other substitutions can also contribute positively. In contrast, the monomeric sesquiterpenoids **5** and **6**, lacking the dimeric scaffold and additional hydroxylation, show no anti‐biofilm activity. These observations suggest that the dimeric scaffold is a critical determinant of bioactivity in aristolane‐type sesquiterpenoids, with hydroxylation and specific substituents further modulating potency. Similar trends in the bioactivities of dimeric and monomeric aristolane‐type sesquiterpenoids have been reported in previous studies [17, 21, 26].

Although compounds **1**‐**4** demonstrated potent anti‐biofilm activity at concentrations as low as 1–2 µg/mL, these values lie relatively close to the IC_50_ ranges observed for a major proportion of mammalian and cancer cell lines used in our cytotoxicity assay (approximately 0.1–1 µg/mL for KB3.1, MCF7, A‐549, and PC‐3). Nonetheless, this limitation does not preclude their potential for topical applications instead of systemic administration, such as wound treatment or device coating, where active concentrations can be applied with minimal systemic exposure.

## 4 Conclusions

In this study, four dimeric aristolane‐type sesquiterpenoids (**1**–**4**), two monomeric aristolane‐type sesquiterpenoids (**5** and **6**), and one benzoate (**7**) were isolated from EtOAC extracts of *N. nambi* cultured in liquid medium. Among these, only the aurisins (**1**–**4**) exhibited significant bioactivity, demonstrating promising antibacterial and antibiofilm activity against *S. aureus*. While their cytotoxicity presents a limitation for systemic therapeutic use, they remain highly relevant for non‐therapeutic applications, such as topical use and surface disinfection [27]. To unlock their full potential, further studies should aim to clarify their mechanisms of action and identify structural features responsible for both bioactivity and toxicity, providing a basis for targeted structural modification.

### 4.1 Experimental

#### 4.1.1 General Experimental Procedure

Analytical HPLC chromatograms and ESI‐MS were acquired using a Thermo‐Fischer Scientific UltiMate 3000 Series UPLC (Waltham, MA, USA) equipped with a C_18_ column (Acquity UPLC BEH 50 × 2.1 mm, 1.7 µm; Waters, Milford, MA, USA) and an amaZon speed ESI‐Iontrap‐MS (Bruker Daltronics, Bremen, Germany) with a sample injection volume of 2 µL and a flow rate of 0.6 mL/min. A gradient elution was applied using a mobile phase consisting of solvent A: H_2_O + 0.1% formic acid (FA, v/v) and solvent B: acetonitrile (ACN) + 0.1% FA (v/v). The gradient started at 5% B for 0.5 min, gradually increasing to 100% B over 20 min, followed by a 10‐min hold at 100% B, with ultraviolet‐visible (UV/Vis) detection in the range of 200–600 nm.

HR‐ESIMS were obtained using an Agilent 1200 Infinity Series HPLC‐UV system (Agilent Technologies, Santa Clara, CA, USA) equipped with a C18 Acquity UPLC BEH analytical column (50 × 2.1 mm, 1.7 µm; Waters, Milford, MA, USA) connected to a maXis ESI‐Quadrupole‐Time‐of‐Flight‐HRMS (ESI‐QTOF‐HRMS; Bruker), scan range 100–2500 *m/z*, set capillary voltage 4500 V, dry temperature 200°C, in positive ionization mode. The experimental conditions for acquiring the HR‐ESI‐MS data were identical to those used for ESI‐MS. Molecular formulas were determined using the Smart Formula algorithm in Compass data analysis software (Bruker, version 6.1).

To compare and characterize the isolated compounds, the UV absorption of those was measured. A UV‐2450 spectrophotometer (Shimadzu Corporation, Kyoto, Japan) was used to measure the UV absorbance properties of the isolated metabolites between 190 and 600 nm at 1 nm intervals to determine the maximum absorbance values of each compound. The compounds were dissolved in CHCl_3_ at a final concentration of 0.1 – 0.05 mg/mL and transferred to a cuvette with a 10 mm path length. The extinction coefficient of the absorbance maxima was calculated using the Beer‐Lambert Law.

Optical activity measurements were conducted using an Anton Paar MCP‐150 circular polarimeter, while UV/Vis spectra were recorded with a Shimadzu UV‐2450 spectrophotometer. CD measurement was performed using a Jasco J‐1500 CD spectrometer, and methanol was used as the solvent. IR spectra were measured using a PerkinElmer Spectrum 100 FT‐IR spectrometer. NMR spectra were recorded using Bruker Avance III spectrometers (500 MHz with BBFO Smart Probe and 700 MHz with TCI cryoprobe) and referenced to chemical shifts of solvent used [CDCl_3_ (^1^H: 7.26 ppm, ^13^C: 77.16 ppm), CD_3_CN (^1^H: 1.94 ppm, ^13^C: 118.26 ppm)], depending on their solubility.

#### 4.1.2 Cultivation, Extraction, and Isolation

The fruiting bodies of *N. nambi* were collected in 2022 from Arabuko Sokoke National Park, Kenya, Africa. Following collection, the strain was isolated, identified through ITS and LSU sequencing, and subsequently deposited in the Czech Collection Clavicipitales (CCC) under the catalog reference CCC 1560. The specimen was identified using the morphology and ITS rDNA sequence (Genbank Accession no. PX735261) generated according to Kolařík et al. [28]. Based on the BlastN similarity search in NCBI GenBank, the ITS barcode has the full identity to *Neonothopanus nambi* entry MK928490.1, from the study of Hu et al. [29].

For scaled‐up cultivation, YM 6.3 medium was selected from a range of media used for small‐scale fermentation. The fungus was initially grown on YM 6.3 agar at 23°C for 4 days. Post‐incubation, the colonies were cut into small pieces (5 mm in diameter) using a cork borer. Four of these pieces were transferred into one 500 mL Erlenmeyer flask containing 200 mL of media and incubated as a preculture at 23°C at 140 rpm shaking. The preculture was incubated until visible growth, homogenized (IKA ULTRA TURRAX Disperser), and 8 mL of culture was distributed into 24 separate 500 mL Erlenmeyer flasks containing 200 mL of media each, yielding a total cultivation volume of 4.8 L, and incubated at 23°C under shaking conditions (140 rpm) till glucose consumption. Glucose levels were monitored daily using Medi‐Test Glucose strips (Machery‐Nagel, Germany) until complete glucose consumption, which took 5 days. Cultures were harvested after another 5 days of glucose consumption and extracted with EtOAc, yielding 996.5 mg of supernatant extract and 586.3 mg of mycelial extract. For extraction, the protocol described previously by Schrey et al. was adopted [30].

For pre‐purification, mycelial and supernatant extracts were fractionated through a solid‐phase extraction (SPE) cartridge (Strata TM‐X 33 µm Polymeric Reversed Phase 1 g/20 mL, Phenomenex, Germany) and separated into six different fractions based on their solubility in non‐polar to polar solvents: 100% H_2_O (24.0 mg supernatant), 20% ACN for only supernatant (228.0 mg), 40% ACN (250.0 mg supernatant, 78.0 mg mycelia), 100% ACN (466.0 mg supernatant, 401.0 mg mycelia), 100% MeOH (4.8 mg supernatant, 66.0 mg mycelia), 100% acetone (22.7 mg supernatant, 40.9 mg mycelia). Analyzed through HPLC‐ESIMS analysis, targeted secondary metabolites appeared in ACN, MeOH, and acetone fractions, which were then subjected to preparative HPLC for isolation. Compounds **1**–**4** appeared in all ACN, MeOH, and acetone fractions, whereas compounds **5**–**7** were detected in 20% and 40% ACN fractions. The extracts were separated using a PLC 2050 preparative HPLC system (Gilson, Middleton, WI, USA), with Nucleodur Phenyl‐Hexyl (250 × 40 mm, 5 µm; Macherey‐Nagel) column as the stationary phase and following conditions as mobile phase: solvent A: deionized H_2_O + 0.1% FA; solvent B: ACN + 0.1% FA; flow: 40 mL/min, conducted with the following elution gradients.

For supernatant and mycelial fractions from SPE‐prepurification with 100% MeOH: solvent B increasing from 20% to 50% B in 15 min, followed by increasing from 50% to 65% B in 32 min, from 65% to 86% B in 7 min, and 86% to 100% B in 11 min. Compound **3** (141.4 mg) was obtained in pure form as a result of this separation with *t*_R_ = 10 min. Compound **4** (1.4 mg) was obtained as a minor yellow colored fraction, not detected by the system's UV detector. However, it was collected, dried, concentrated and analyzed through LCMS, leading to the identification of aurisin G (**4**). Both supernatant fractions of SPE‐prepurification with 40% and 100% ACN: solvent B increasing from 10% to 40% in 15 min, increasing from 40% to 55% B in 20 min, isocratic conditions at 55% B for 20 min, and followed by increasing from 55% to 100% B in 10 min. Mycelial fraction from SPE‐prepurification with 40% ACN: Solvent B increasing from 30% to 40% in 10 min, 40% to 50% in 20 min, 50% to 65% in 15 min, 65% to 70% in 10 min, 70% to 100% in 20 min, and followed by isocratic conditions at 100% for 5 min. Mycelial fraction from SPE‐prepurification with 100% ACN: Solvent B increasing from 20% to 45% in 18 min 20 s, followed by isocratic conditions at 45% for 41 min 40 s and increasing from 45% to 95% in 6 min. For the supernatant fraction from SPE‐prepurification with 20% ACN, size‐exclusion chromatography using ÄKTA pure Cytiva OptiRun was performed. A Glass column filled with Sephadex LH20, with 100% MeOH and 0.1% FA as the solvent and a flow rate of 3 mL/min, was employed. The targeted compounds appeared in the fraction with retention time *t*_R_ = 17.0–18.5 min. Compounds **1** and **2** and **5**–**7** were isolated through semi‐preparative RP‐HPLC (Agilent Technologies 1200 series) using X‐Bridge C18 (250 × 10 mm, 5 µm; Waters) column as the stationary phase and following varying conditions as mobile phase. Compounds **1** and **2**: solvent A: deionized H_2_O, solvent B: ACN; conducted under isocratic conditions at 39% B. This resulted in purification of compounds **1** (88.9 mg) and **2** (15.7 mg) with retention times *t*_R_ = 34–38 and 31.0–32.5 min, respectively. Compound **5**: solvent A: deionized H_2_O + 0.1% FA, solvent B: ACN + 0.1% FA, conducted under isocratic conditions at 30% B. This led to the isolation of compound **5** (2.3 mg) with *t*_R_ = 9 min. Compound **6**: solvent A: deionized H_2_O, solvent B: ACN, conducted under the isocratic conditions at 35% B. This led to the isolation of compound **6** (1.1 mg) with *t*_R_ = 16.5–17.75 min. Compound **7**: solvent A: deionized H_2_O, solvent B: ACN; conducted under isocratic conditions at 35% B, which led to the isolation of compound **7** (2.2 mg) with *t*_R_ = 17.0–18.5 min.

**Aurisin D (1)**: Light yellow powder; ${\left[\alpha \right]}_{D}^{20}$ +167.6 (*c* 1.0, CHCl_3_); UV/Vis (CHCl_3_): λ_max_ (log ε) = 198 (0.57), 236.5 (0.15), 308 (0.56) nm; CD (MeOH): λ (Δε) = 201 (−3.88), 214 (−1.14), 226 (−3.20), 262 (5.06), 312 (−5.29), 355 (7.93) nm; ESI‐MS: *m*/*z* 526.29 [M–H_2_O + H]^+^, *m*/*z* 541.25 [M–H]^−^; HR‐ESIMS: *m*/*z* 543.2585 [M + H]^+^ (calcd. 543.2585 for C_30_H_39_O_9_^+^), *t*_R_ = 9.72 min (LC‐ESI‐MS). For ^1^H and ^13^C NMR data, see Table 1.

**Aurisin B (2)**: White powder; ${\left[\alpha \right]}_{D}^{20}$ = +16.7 (c 1.0, CHCl_3_); UV/Vis (CHCl_3_): λ_max_ (log ε) = 226.50 (0.078), 299 (0.966) nm; CD (MeOH): λ (Δε) = 199 (−4.66), 215 (−0.78), 227 (−1.62), 263 (0.56), 285 (0.30), 307 (1.00), 325 (0.38), 348 (2.01) nm; ESI‐MS: *m*/*z* 567.31 [M + Na]^+^, 543.31 [M–H]^−^; HR‐ESIMS: *m/z* 545.2761 [M + H]^+^, (calc. 545.2761 for C_30_H_41_O_9_^+^) *t*_R_ = 9.59 min (LC‐ESI‐MS). For ^1^H and ^13^C NMR data, see Table 1.

#### 4.1.3 Antibacterial and Cytotoxicity Assays

All compounds isolated in this study were evaluated for their antimicrobial activity through a serial dilution assay, with concentrations ranging from 66 to 0.5 µg/mL. The experimental design was carried out according to established protocols [31]. The MIC was determined against a broad spectrum of pathogens, including five fungal species: *Candida albicans* (DSM 1665), *Mucor hiemalis* (DSM 2656), *Schizosaccharomyces pombe* (DSM 70572), *Rhodotorula glutinis* (DSM 10134), and *Wickerhamomyces anomalus* (DSM 6766). Additionally, the assay encompassed several Gram‐positive bacteria, such as *Staphylococcus aureus* (DSM 346), *Bacillus subtilis* (DSM 10), and *Mycobacterium smegmatis* (ATCC 700084), as well as Gram‐negative bacteria, including *Acinetobacter baumannii* (DSM 30008), *Escherichia coli* (DSM 1116), *Chromobacterium violaceum* (DSM 30191), and *Pseudomonas aeruginosa* (PA14). Gentamicin and nystatin were used as positive controls for bacterial and fungal strains, respectively. For specific pathogens (*A. baumannii, B. subtilis*, and *M. smegmatis*), ciprofloxacin, oxytetracycline, and kanamycin were employed as positive controls (Table S4) [32].

The cytotoxic potential of the isolated compounds was evaluated using the MTT assay, spanning concentrations from 1 to 37 µg/mL, against two mammalian cell lines: mouse fibroblasts (L‐929) and human endocervical adenocarcinoma (KB‐3.1), additional cancer cell lines, including prostate cancer (PC‐3), breast cancer (MCF‐7), ovarian cancer (SKOV‐3), epidermoid carcinoma (A431), and lung cancer (A‐549) following a previously established protocol [31]. Epothilone B was used as the positive control (Table S4). After a 5‐day incubation period, the minimum concentration required to inhibit 50% of cell growth (IC_50_ values) was determined. Experiments were performed in two independent biological replicates.

#### 4.1.4 Biofilm Inhibition Assay

The biofilm inhibition activity of compounds **1**–**7** was assessed against *S. aureus* (DSM 1104) following the previously established protocol with minor changes [33]. In brief, *S. aureus* was cultured and adjusted to a 0.001 McFarland standard, then incubated with serial dilutions of the compounds (66–0.5 µg/mL) in 96‐well plates (TPP Tissue Culture, ref no. 92196) for 24 h. Biofilm inhibition was quantified using crystal violet (CV) staining, with methanol serving as the negative control and microporenic acid A (MAA) as the positive control [34]. Data are expressed as means ± standard deviation (SD), with duplicates in two biological repeats (n = 4). For the biofilm eradication assay, *S. aureus* was cultured and adjusted to a 0.01 McFarland standard, and incubated for 24 h to establish biofilms prior to the treatment with serial dilutions of the compounds (66–0.5 µg/mL) in 96‐well plates (TPP Tissue Culture, ref no. 92196), for another 24 h. Experiments were performed in three independent biological replicates.

#### 4.1.5 Confocal Time‐Lapse Analysis of Drug Treatment on Established *S. aureus* Biofilms

An overnight fresh culture of *S. aureus* USA300 was diluted to 0.01 McFarland standard in CASO medium with 0.4% glucose and cultured in a microtiter plate (Greiner Bio‐One, Kremsmünster, Austria), covered with an air‐permeable breath seal cover foil (Greiner Bio‐One). The Biofilms were allowed to establish for 24 h at 37°C in static conditions. Subsequently, the biofilm wells were treated with compounds **1** and **2** at two specific concentrations, i.e., 62 µg/mL and 8 µg/mL, in three technical repeats. 2% Methanol was used as a solvent control, and 25% ethanol was used as a positive, killing control. Staining was performed with BacLight Live/Dead Kit (Thermo Fisher Scientific, Massachusetts, USA), utilizing SYTO 9 to label viable cells and propidium iodide to identify cells with compromised membranes. Stained biofilms were subjected to time‐lapse CLSM imaging using an inverted SP8 microscope (Leica Microsystems, Wetzlar, Germany) operated with LAS X software. Images were collected over a 24‐h period with two settings: an overview z‐stack (height: 40 µm, step size: 2 µm; 1024 × 1024 pixel; pixel size: 0.378 µm) to calculate the overall biofilm biovolume and a zoom z‐stack (height: 40 µm, step size: 2 µm; 512 × 512 pixel; pixel size: 0.071 µm) to calculate the percentage of green and red biofilm bacteria [35]. CLSM images of both biofilm stacks were analyzed using Developer XD (Definiens, München, Germany) to quantify the above‐mentioned parameters, including red, green, and total biovolume, and the alive and dead biovolume.

#### 4.1.6 Statistical Analysis

The difference between the treatment groups and the control group was evaluated using one‐way analysis of variance followed by Dunnett's post‐hoc test. Statistical significance was defined as *p* < 0.05. All analyses were performed using GraphPad Prism 9 (GraphPad Software, San Diego, CA, USA).

## Author Contributions

**Syeda Javariya Khalid**: data curation, formal analysis, and writing – original draft. **Yuanyuyue Huang**: data curation, formal analysis, methodology, and writing – original draft. **Mark Kimani Njogu**: data curation, investigation, formal analysis, and writing – original draft. **Mathias Müsken**: data curation, investigation, supervision, and formal analysis. **Miroslav Kolařík**: data curation, formal analysis, methodology, and writing – original draft. **Hedda Schrey**: conceptualization, project administration, supervision, formal analysis, writing, and review and editing.

## Conflicts of Interest

The authors declare no conflicts of interest.

## Supporting information




**Supporting file 1**: cbdv70882‐sup‐0001‐SuppMat.docx.

## Data Availability

The authors have nothing to report.
