# Supplementary material for: Targeting Staphylococcus aureus Biofilms Through Aurisin Derivatives From Neonothopanus nambi of African Origin
Source: Chem Biodivers. 2026 Feb 3;23(2):e03741. doi: 10.1002/cbdv.202503741 (PMC12866744; doi:10.1002/cbdv.202503741)
Supplement: Supplementary file 1 — Supporting file 1: cbdv70882‐sup‐0001‐SuppMat.docx. [file CBDV-23-e03741-s001.docx]

**Supplementary material**

**Targeting *Staphylococcus aureus* Biofilms through Aurisin Derivatives from *Neonothopanus nambi* of African Origin**

Syeda J. Khalid,^a, b^ Njogu M. Kimani, ^c, d^ Yuanyuyue Huang,^a, b^ Mathias Müsken,^e^ Jan-Peer Wennrich,^a, b^ Jonas Bentrup, ^f^ Miroslav Kolařík,^g, h^ Josphat C. Matasyoh,^i^ and Hedda Schrey*^,a^

^a^Department of Microbial Drugs, Helmholtz Centre for Infection Research GmbH (HZI) and German Centre for Infection Research (DZIF), Inhoffenstrasse 7, 38124 Braunschweig, Germany

^b^ Institute of Microbiology, Technische Universität Braunschweig, Spielmannstrasse 7, 38106 Braunschweig, Germany

^c^ Institute for Organic and Analytical Chemistry, University of Bremen, Leobener Strasse 7, 28359 Bremen, Germany

^d^ Department of Physical Sciences, University of Embu, Embu 6-60100, Kenya

^e^ Central Facility for Microscopy, Helmholtz Centre for Infection Research GmbH (HZI) and German Centre for Infection Research (DZIF), Inhoffenstrasse 7, 38124 Braunschweig, Germany

^f^ Institute for Physical and Theoretical Chemistry, University of Bremen, Bremen, Germany

^g^ Institute of Microbiology, Academy of Sciences of the Czech Republic, Prague, Czech Republic ^g^ Department of Botany, Faculty of Science, Charles University, Prague, Czech Republic

^h^ Department of Botany, Faculty of Science, Charles University, Prague, Czech Republic

^i^ Department of Chemistry, Egerton University, Njoro, Kenya

*Corresponding author: hedda.schrey@helmholtz-hzi.de Tel.: +49-531-6181-4239

**Table of content.**

| # | Contents | Page |
| --- | --- | --- |
| 1  2  3  4  5  6  7  8  9  10  11  12  13  14  15  16  17  18  19  20  21  22  23  24  25  26  27  28  29  30  31  32  33  34  35  36  37  38  39  40  41 | Table S1. Media composition  Table S2. Biofilm inhibition of *S. aureus* by compounds **1**-**4**  Table S3. Biofilm eradication of *S. aureus* by compounds **1**-**4**  Table S4. MIC and cytotoxicity assays of compounds **1**-**4**  Figure S5. LR-ESIMS spectrum of **1**  Figure S6. HR-ESIMS of compound **1**  Figure S7. IR spectrum of compound **1**  Figure S8. UV spectra of compound **1** in chloroform 0.05 mg/mL.  Figure S9. ECD spectra of compound **1**.  Figure S10. ^1^H NMR Spectrum for compound **1** (500 MHz) in CD_3_CN  Figure S11. ^13^C NMR Spectrum for compound **1** (125 MHz) in CD_3_CN  Figure S12. COSY NMR spectrum for compound **1** (500 MHz) in CD_3_CN  Figure S13. HMBC NMR spectrum for compound **1** (500 MHz) in CD_3_CN  Figure S14. HSQC NMR spectrum for compound **1** (500 MHz) in CD_3_CN  Figure S15. NOESY NMR spectrum for compound **1** (500 MHz) IN CD_3_CN  Figure S16. LR-ESI-MS spectrum of compound **2**  Figure S17. HR-ESI-MS spectrum of compound **2**  Figure S18. IR spectrum of compound **2**  Figure S19. UV spectra of compound **2** in chloroform at the concentration of 0.05 mg/mL  Figure S20. ECD spectra of compound **2**  Figure S21. ^1^H NMR Spectrum for compound **2** (500 MHz) in CDCl_3_  Figure S22. ^13^C NMR Spectrum for compound **2** (125 MHz) in CDCl_3_  Figure S23. COSY NMR spectrum for compound **2** (500 MHz) in CDCl_3_  Figure S24. HMBC NMR spectrum for compound **2** (500 MHz) in CDCl_3_  Figure S25. HSQC NMR spectrum for compound **2** (500 MHz) in CDCl_3_  Figure S26. NOESY NMR spectrum for compound **2** (500 MHz) in CDCl_3_  Figure S27. HR-ESIMS spectrum of compound **3**  Figure S28. ^1^H NMR spectrum for compound **3** (500 MHz) in CD_3_Cl  Figure S29. ECD spectra of compound **3**  Figure S30. LR-ESIMS spectrum of compound **4**  Figure S31. HR-ESIMS spectrum of compound **4**  Figure S32. ^1^H NMR Spectrum for compound **4** (500 MHz) in CD_3_CN  Figure S33. ^13^C NMR Spectrum for compound **4** (125 MHz) in CD_3_CN  Figure S34. ECD spectra of compound **4**  Figure S35. ECD spectra of compounds **1**-**4**  Figure S36. HR-ESIMS spectrum for compound **5**  Figure S37. ^1^H MNR spectrum for compound **5** (500 MHz) IN CD_3_CN  Figure S38. HR-ESIMS spectrum of compound **6**  Figure S39. ^1^H NMR spectrum of compound **6** (500 MHz) in CD_3_CN  Figure S40. HR-ESIMS spectrum of compound **7**  Figure S41. ^1^H NMR Spectrum for compound **7** (500 MHz) in MeOD | 3  4  4  5  6  7  8  8  9  10  10  11  11  12  12  13  14  15  16  16  17  18  18  19  20  21  22  23  23  24  25  26  26  27  27  28  29  30  31  32  33 |

**Table S1: Media composition.**

| **Media** | **Ingredients** | **Concentration [g/L]** | **Manufacturer** | **Type** | **pH value** |
| --- | --- | --- | --- | --- | --- |
| Q6/2  (Cottonseed Medium) | **Cotton seed flour**  **D-Glucose**  **Glycerol** | **5**  **2.5**  **10** | Sigma-Aldrich Chemie GmbH;  Steinheim am Albuch, Germany  Cerestar Deutschland GmbH;  Krefeld, Germany  Carl Roth GmbH & Co. KG; Karlsruhe, Germany | **Liquid** | **7.2** |
| YM6.3  (Yeast Malt Medium) | D-glucose  Malt extract  Yeast extract | 4  10  4 | Cerestar, Deurotschland GmbH  Gibco, Thermo Fisher Scientific  Inc.; Ma, USA  Herrnmühle; Reichelsheim, Germany | Liquid | 6.3 |
| YM6.3 agar | D-glucose  Malt extract  Yeast extract  Agar | 4  10  4  20 | Cerestar, Deurotschland GmbH  Gibco, Thermo Fisher Scientific  Inc.; Ma, USA  Herrnmühle; Reichelsheim, Germany  Becton, Dickinson  and Company;  Sparks, USA | Solid | 6.3 |
| ZM/2  (Sugar Malt Medium) | Yeast extract  D-glucose  Edamine  Mannitol  Molasses  (NH_4_)_2_SO_4_  Oatmeal  Sucrose | 4  1.5  0.5  4  5  0.5  5  4 | Herrnmühle; Reichelsheim, Germany  Cerestar Deutschland GmbH;  Krefeld, Germany  Thermo Fisher Scientific Oxoid  Ltd.; Hampshire, UK  AppliChem GmbH; Darmstadt,  Germany  Nordzucker AG; Braunschweig,  Germany  Merck KGaA; Darmstadt, Germany  Herrnmühle; Reichelsheim, Germany  Carl Roth GmbH & Co. KG; Karlsruhe,  Germany |  |  |
| CM  (Cornmeal-Medium) | Cornmeal  Glucose  Yeast extract | 20  4  1 | Frießinger Mühle GmbH, Bad Wimpfen, Germany  Cerestar Deutschland GmbH;  Krefeld, Germany  Herrnmühle; Reichelsheim, Germany | Liquid | 5.5 |
| SYM | Malt extract  Saccharose  Yeast extract | 30  10  5 | Gibco, Thermo Fisher Scientific  Inc.; Ma, USA  Organotechnie; La Courneuve France | Liquid | 6.3 |
| SNL | D(+)-Glucose-monohydrate  L-Asparagine-monohydrate  KH2PO4  MgSO4 x 7H2O  Yeast extract  Trace Element Solution | 30  4.5  1.5  0.5  3  1 mL | Cerestar Deutschland GmbH; Krefeld, Germany  SERVA Electrophoresis GmbH, Heideberg, Germany  Carl Roth GmbH & Co. KG; Karlsruhe, Germany  Merck KgaA, Darmstadt, Germany  Organotechnie; La Courneuve  France  / | Liquid | 6 |
| Trace element solution | FeCl_3_ x 6H_2_O  ZnSO_4_ x 7H_2_O  MnSO_4_ x H_2_O  CuSO_4_ x 5H_2_O  EDTA | 0.08  0.09  0.03  0.005  0.4 | Merck KgaA, Darmstadt, Germany  MP Biomedicals  Merck KgaA, Darmstadt, Germany  Merck KgaA, Darmstadt, Germany  Sigma Medical Technology GmbH, Steinbach (Taunus), Germany | Liquid |  |
| GDYP | Dextrin  Glucose  Yeast extract  Soy peptone  KH_2_PO_4_  FeCl_3_ x 6 H_2_O  MgSO_4_ x 7H_2_O | 40  10  4  2  2  2  0.5 | Cal Roth GmbH + Co. KG; Kalsruhe, Germany  Cerestar Deutschland GmbH; Krefeld, Germany  Organotechnie; La Courneuve France  Becton Dickinson and Company, le Pont de Claix, France  Carl Roth GmbH & Co. KG; Karlsruhe, Germany  Merck KgaA, Darmstadt, Germany  Merck KgaA, Darmstadt, Germany | Liquid | 3 |
| Rice medium | Rice  Distilled water | 0.1  200mL | Oryza Ideal Reis Langkorn parboiled (500 g), Euryza GmbH, Hamburg, Germany | Solid |  |
| CASO | Peptones from casein  Peptones from soymeal  NaCl | 15  5  5 | Sigma-Aldrich GmbH  Sigma-Aldrich GmbH  Thermo Fisher Scientific; Fair Lawn, USA | Liquid | 7.3 |
| 10X PBS | NaCl  KCl  K_2_HPO_4_  Na_2_HPO_4_ | 100  2.5  3  18 | Thermo Fisher Scientific; Fair Lawn, USA  Avantor-Leistungsmaterialien; Griesheim, Germany  Sigma Medical Technology GmbH, Steinbach (Taunus), Germany | Liquid | 7.4 |

**Table S2: Biofilm inhibition of *S. aureus* by compounds 1-4.**

| ***Staphylococcus aureus* DSM 1104 biofilm inhibition (%±SD)** | | | | | |  |  |  |
| --- | --- | --- | --- | --- | --- | --- | --- | --- |
| **Tested Compound** | **Conc. (µg/ml)** | | | | |  |  |  |
|  | 66 | 33 | 16.5 | 8.25 | 4.125 | 2 | 1 | 0.5 |
| Aurisin D (**1**) | 84 ± 8 | 85 ± 7 | 85 ± 8 | 86 ± 7 | 86 ± 7 | 80 ± 15 | 43 ± 16 | 37 ± 9 |
| Aurisin B (**2**) | 80 ± 10 | 82 ± 9 | 83 ± 9 | 85 ± 7 | 86 ± 7 | 86 ± 7 | 77 ± 15 | 38 ± 5 |
| Aurisin A (**3**) | 80 ± 4 | 81 ± 4 | 81 ± 5 | 80 ± 6 | 79 ± 3 | 44 ± 24 | 11 ± 14 | - |
| Aurisin G (**4**) | 84 ± 3 | 87 ± 2 | 88 ± 2 | 88 ± 3 | 84 ± 6 | 83 ± 9 | 39 ± 17 | - |
| Microporenic acid A (MAA) | 78 ± 11 | - | - | - | - |  |  |  |

**Table S3: Biofilm eradication of *S. aureus* by compounds 1 and 2.**

| ***Staphylococcus aureus* DSM 1104 biofilm eradication (%±SD)** |  | |
| --- | --- | --- |
| **Tested Compound** | **Conc. (µg/ml)** | |
|  | 125 | 62.5 |
| Aurisin D (**1**) | 54 ± 13 | 50 ± 9 |
| Aurisin B (**2**) | 57 ± 4 | 49 ± 9 |
| Microporenic acid A (MAA) | 52 ± 16 | - |

**Table S4: Cytotoxicity (IC_50_) and antimicrobial activity (MIC) of 1-7.**

| **Tested organisms / Cells** **1 2 3 4 5 6 7**  **Epothilone B** | | | | | | | | |
| --- | --- | --- | --- | --- | --- | --- | --- | --- |
| **IC _50_ against mammalian cell lines (μg/mL)** | | | | | | | | |
| Human endocervical adenocarcinoma (KB-3.1) | 0.51 | 0.33 | 0.25 | 0.27 | n.a | n.a | n.a | 0.000058 |
| Mouse fibroblast (L-929) | 22.0 | 7 | 22 | 6.8 | n.i | n.i | n.i | 0.00090 |
| Human epidermoid carcinoma (A-431) | n.a | n.a | n.a | n.d | n.a | n.a | n.a | 0.00023 |
| Human breast adenocarcinoma (MCF-7) | 0.13 | 0.1 | 0.17 | 0.27 | n.i | n.i | 23.0 | 0.00014 |
| Human ovarian cancer (SKOV-3) | n.a | n.a | n.a | n.d | n.a | n.a | n.a | 0.00024 |
| Human lung carcinoma (A-549) | 1.8 | 1.3 | 1.8 | 1.5 | n.a | n.a | n.a | 0.000027 |
| Human prostate carcinoma (PC-3) | 0.6 | 0.72 | 1.3 | n.d | n.a | n.a | n.a | 0.00012 |
| **MIC against fungi (μg/mL)** | | | | | | | | |
| *Pichia anomala* (DSM 6766) | n.i | n.i | n.i | n.d | n.i | n.i | n.i | 4.2^N^ |
| *Schizosaccharomyces pombe* (DSM 70572) | n.i | n.i | n.i | n.d | n.i | n.i | n.i | 4.2^N^ |
| *Candida albicans* (DSM 1665) | n.i | n.i | n.i | n.i | n.i | n.i | n.i | 2.1^N^ |
| *Mucor hiemalis* (DSM 2656) | n.i | n.i | n.i | n.d | n.i | n.i | n.i | 2.1^N^ |
| *Rhodotorula glutinis* (DSM 10134) | n.i | n.i | n.i | n.d | n.i | n.i | n.i | 1.0^N^ |
| **MIC against bacteria (μg/mL)** | | | | | | | | |
| *Escherichia coli* (DSM 1116) | n.i | n.i | n.i | n.d | n.i | n.i | n.i | 0.42^G^ |
| *Bacillus subtilis* (DSM 10) | 2.0 | 2.0 | 4.1 | n.d | n.i | n.i | n.i | 16.6^O^ |
| *Staphylococcus aureus* (DSM 346) | 16.6 | 8.3 | 8.3 | 8.3 | n.i | n.i | n.i | 0.42^G^ |
| *Mycobacterium smegmatis* (ATCC 700084) | n.i | n.i | n.i | n.d | n.i | n.i | n.i | 0.1^K^ |
| *Pseudomonas aeruginosa* (PA14) | n.i | n.i | n.i | n.i | n.i | n.i | n.i | 0.21^G^ |
| *Chromobacterium violaceum* (DSM 30191) | n.i | 66.6 | n.i | n.d | n.i | n.i | n.i | 0.83^G^ |
| *Acinetobacter baumannii* (DSM 30008) | n.i | n.i | n.i | n.d | n.i | n.i | n.i | 0.53^C^ |

n.a: no activity, n.i: no inhibition up to 37 µg/mL for IC_50_ and 66.6 µg/mL for MIC, n.d: not determined, G: gentamycin, O: oxytetracycline, N: nystatin, C: ciprofloxacin, K: kanamycin.

**Figure S5. LR-ESIMS spectrum of compound 1.**

**Figure S6. HR-ESIMS spectrum of compound 1.**


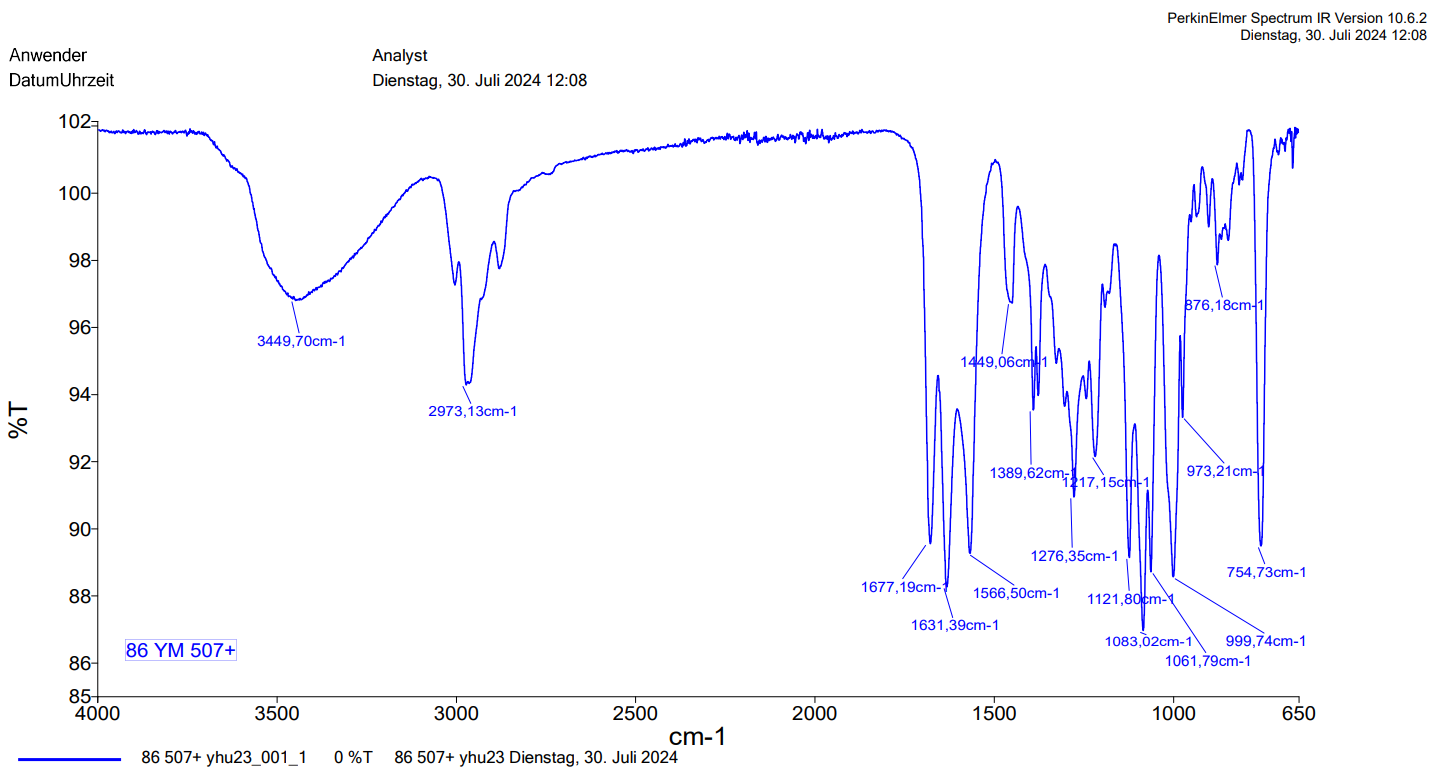


**Figure S7. IR spectrum of compound 1.**


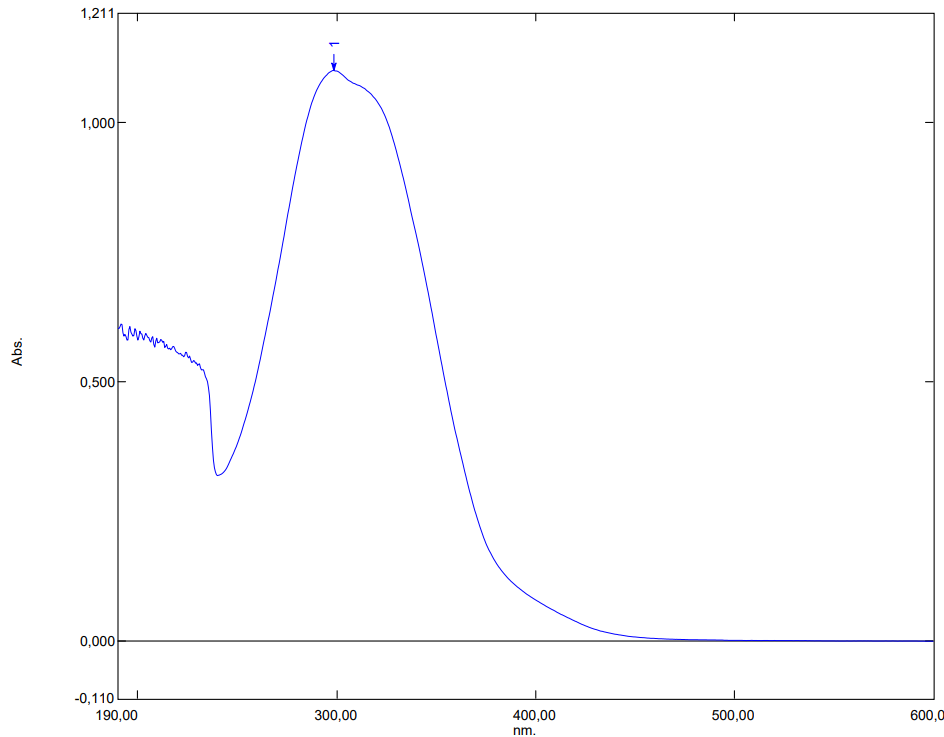


**Figure S8. UV spectra of compound 1 in chloroform at the concentration of 0.05 mg/mL.**


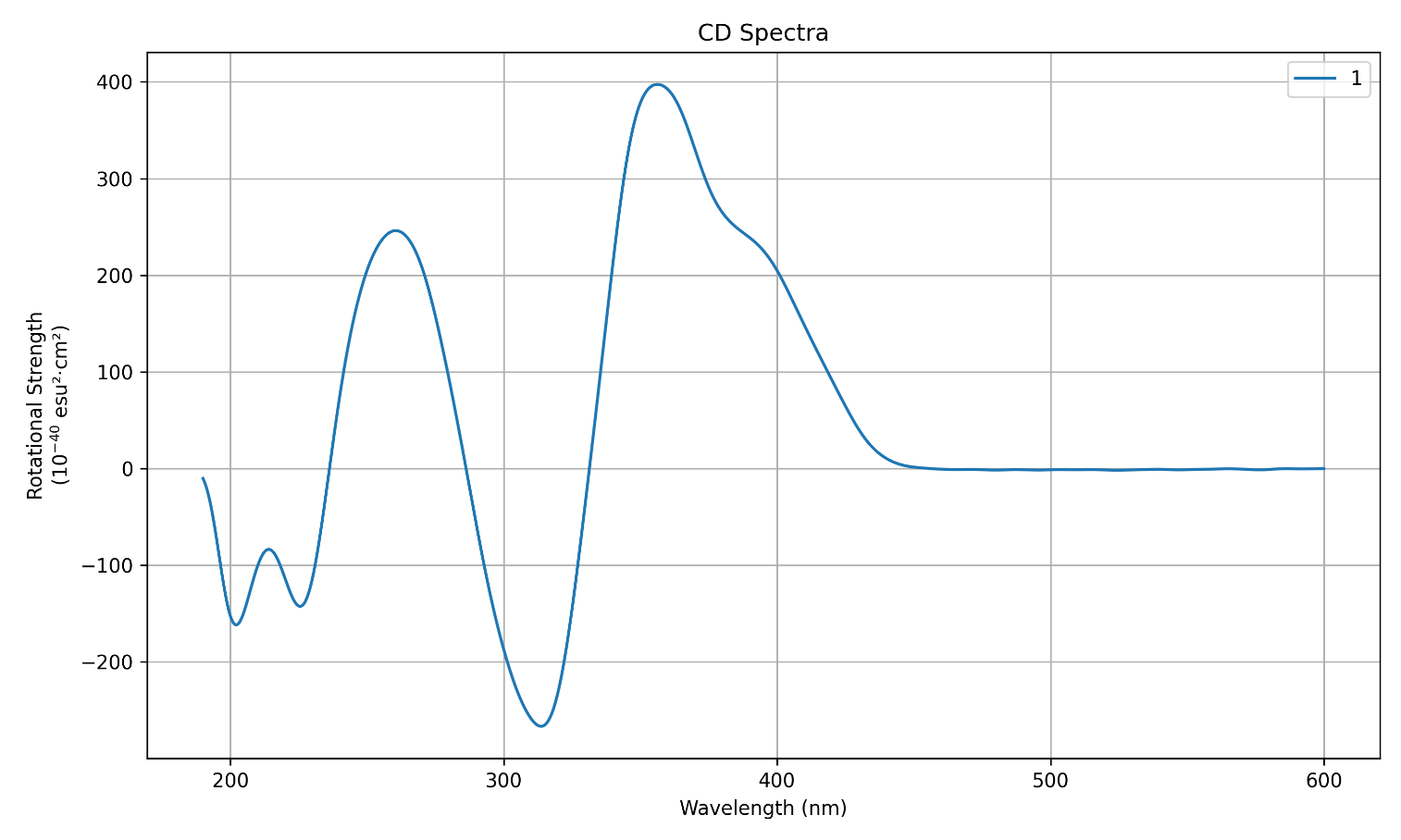


**Figure S9. ECD spectrum of compound 1.**


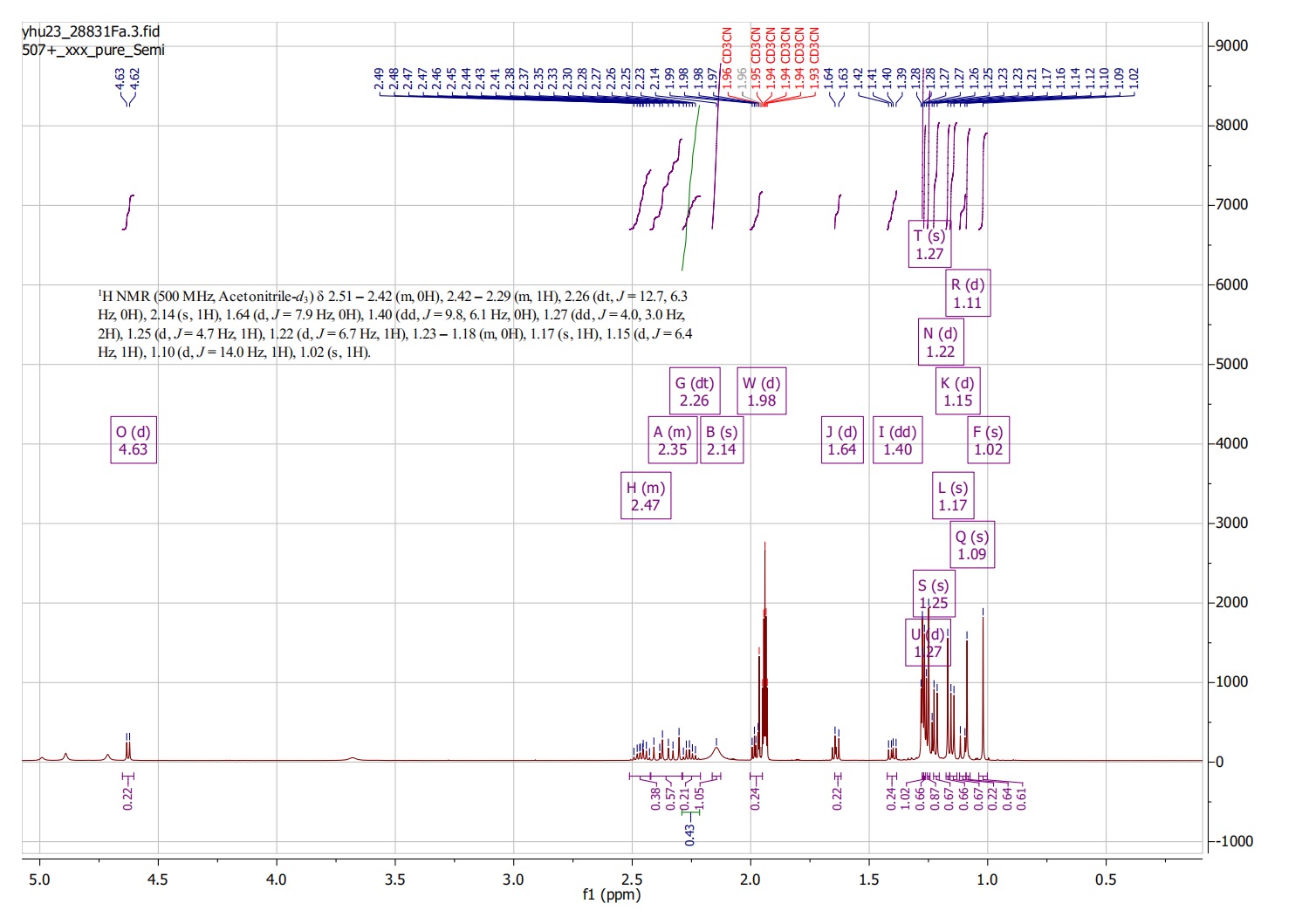


**Figure S10. ^1^H NMR Spectrum for compound 1 (500 MHz) in CD_3_CN.**


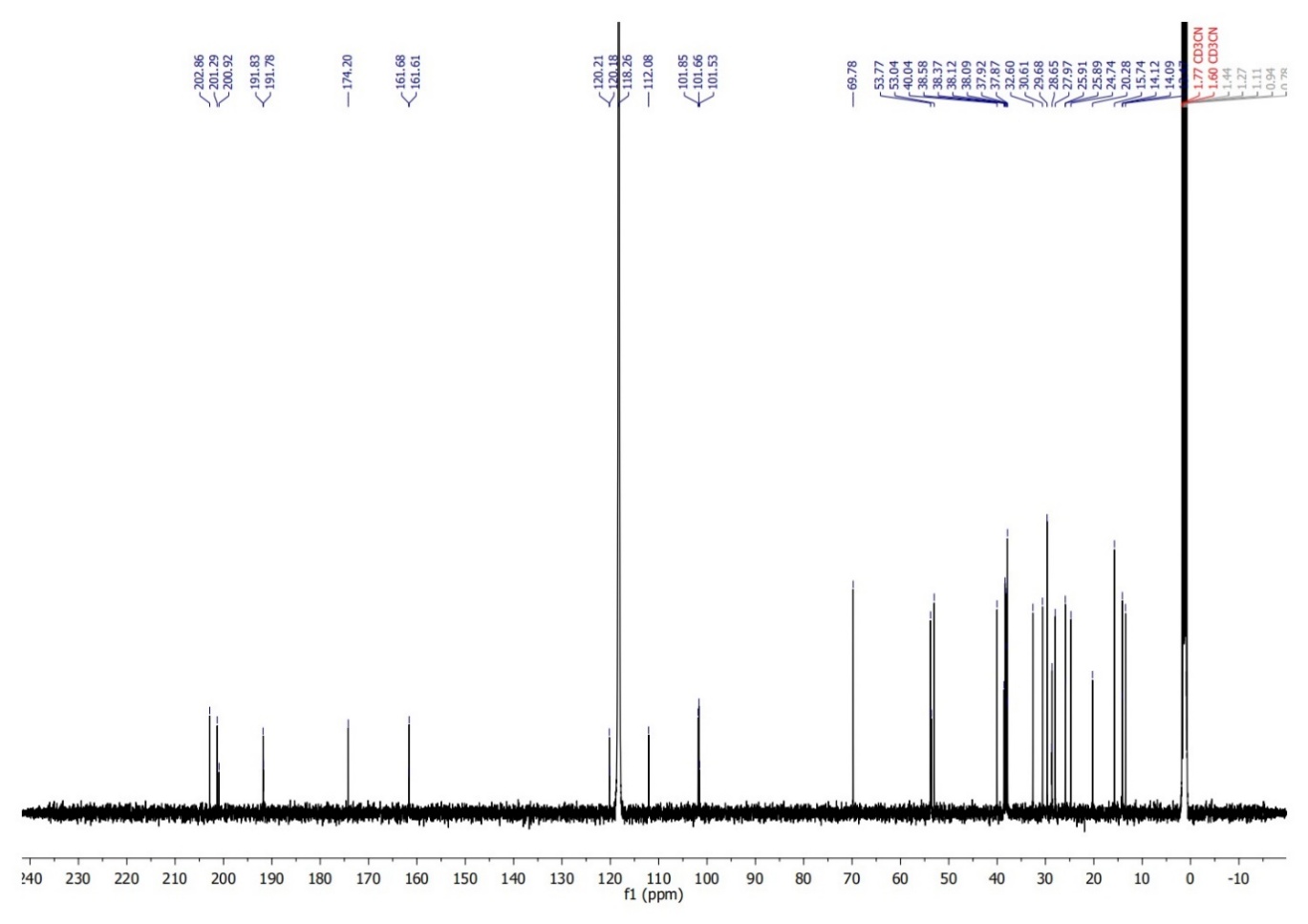


**Figure S11. ^13^C NMR Spectrum for compound 1 (125 MHz) in CD_3_CN.**


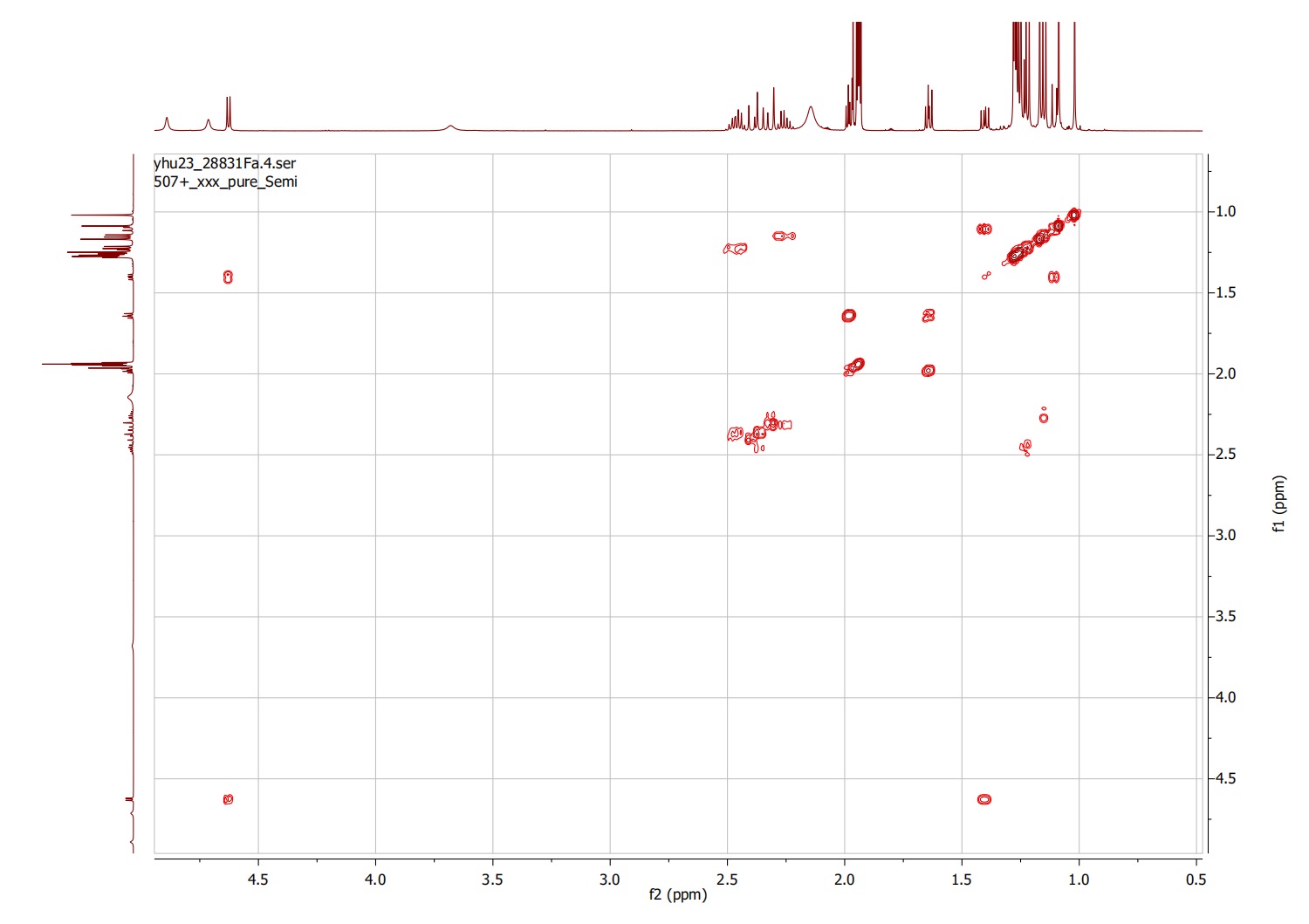


**Figure S12. ^1^H-^1^H COSY spectrum for compound 1 (500 MHz) in CD_3_CN.**


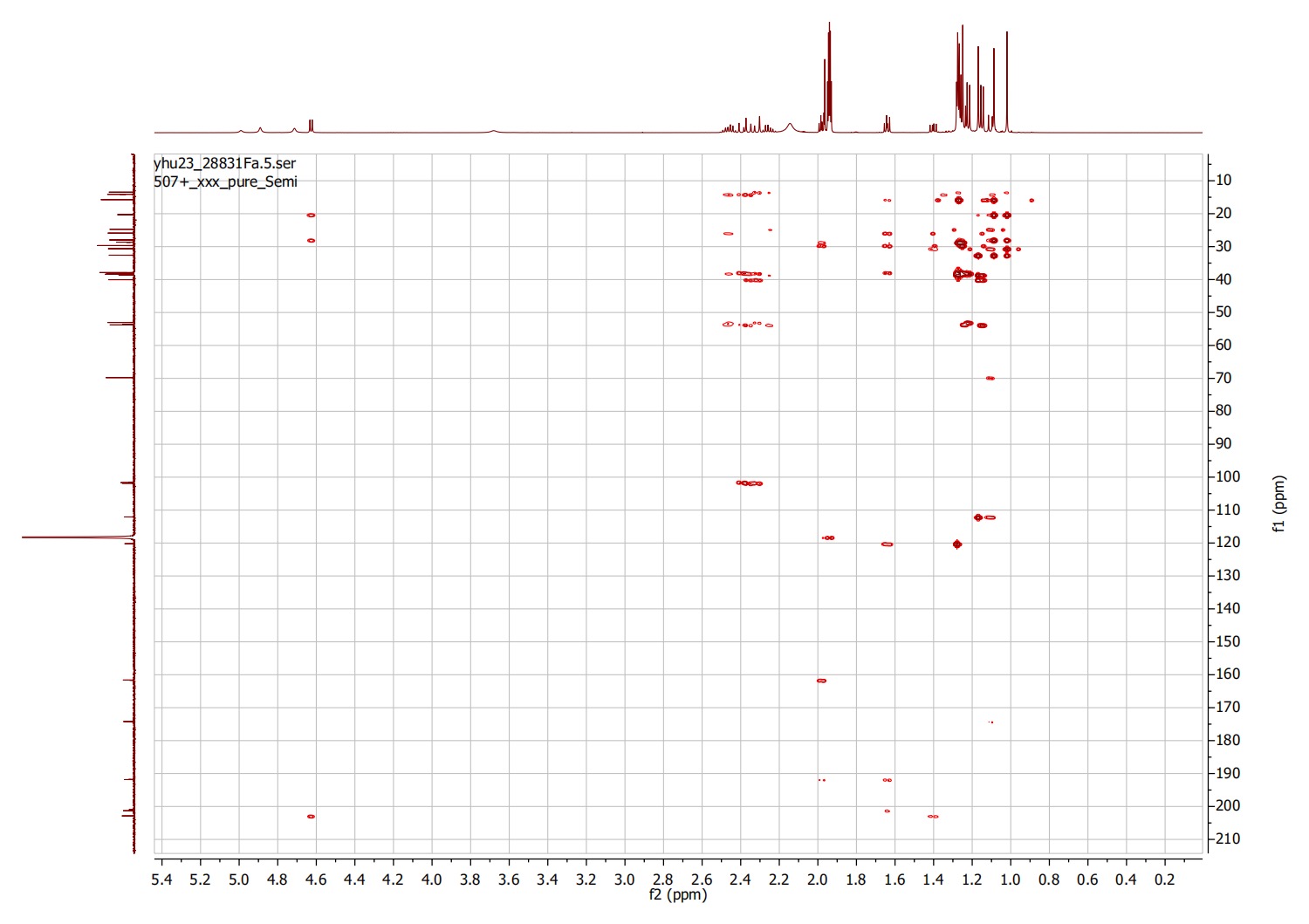
**Figure S13. ^1^H-^13^C HMBC spectrum for compound 1 (500 MHz) in CD_3_CN.**


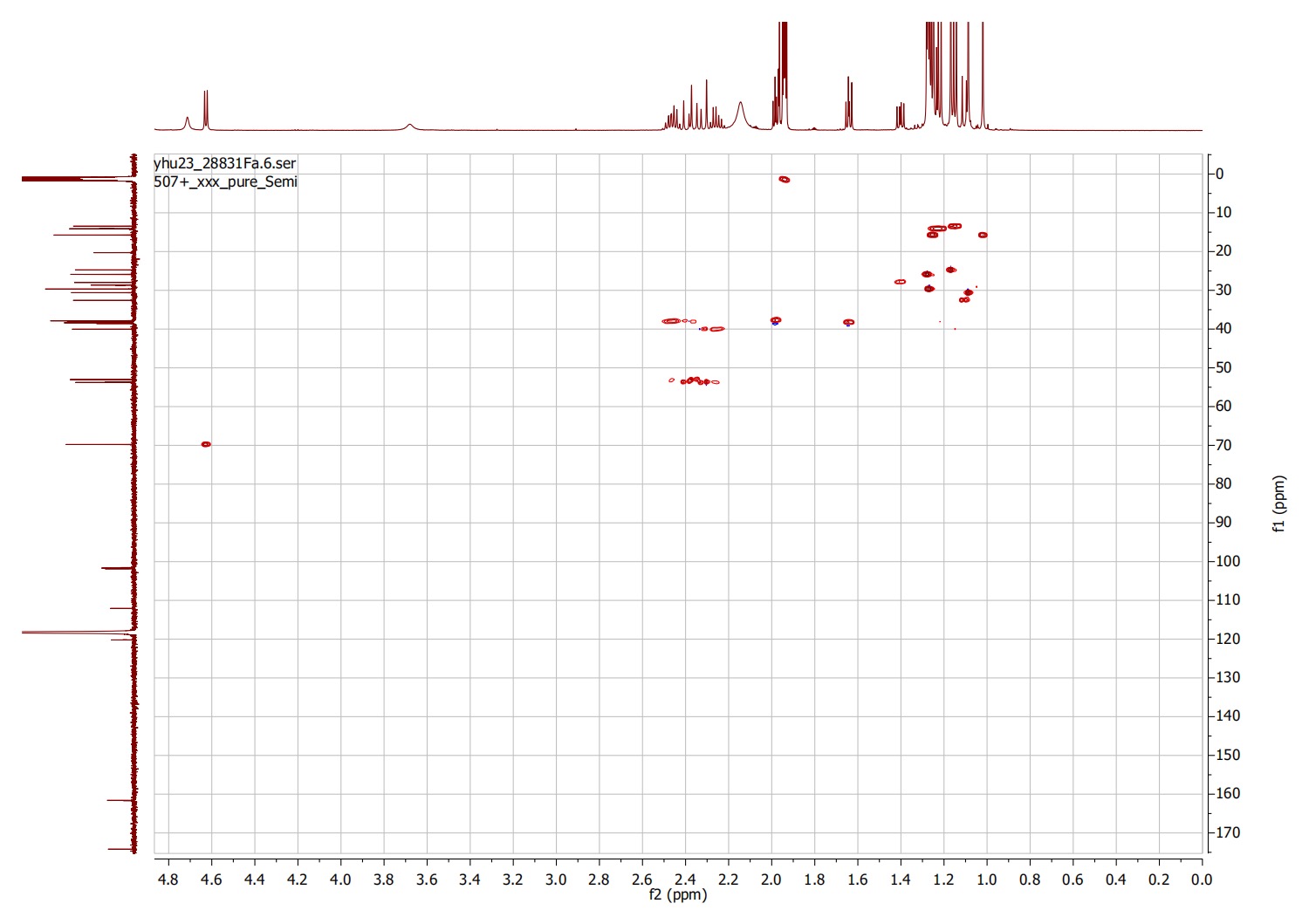


**Figure S14. HSQC spectrum for compound 1 (500 MHz) in CD_3_CN.**

**Figure S15. ^1^H-^1^H NOESY spectrum for compound 1 (500 MHz) in CD_3_CN.**

**Figure S16. LR-ESIMS spectrum of compound 2.**

**Figure S17. HR-ESI-MS spectrum of compound 2.**


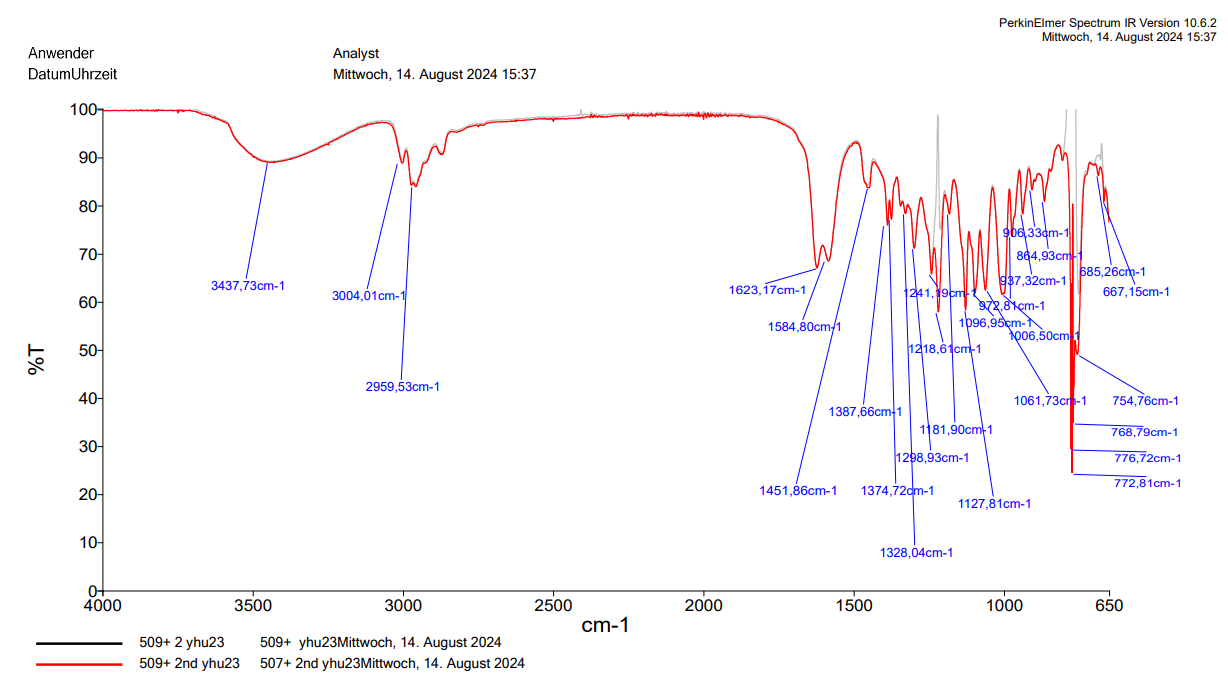


**Figure S18. IR spectrum of compound 2.**


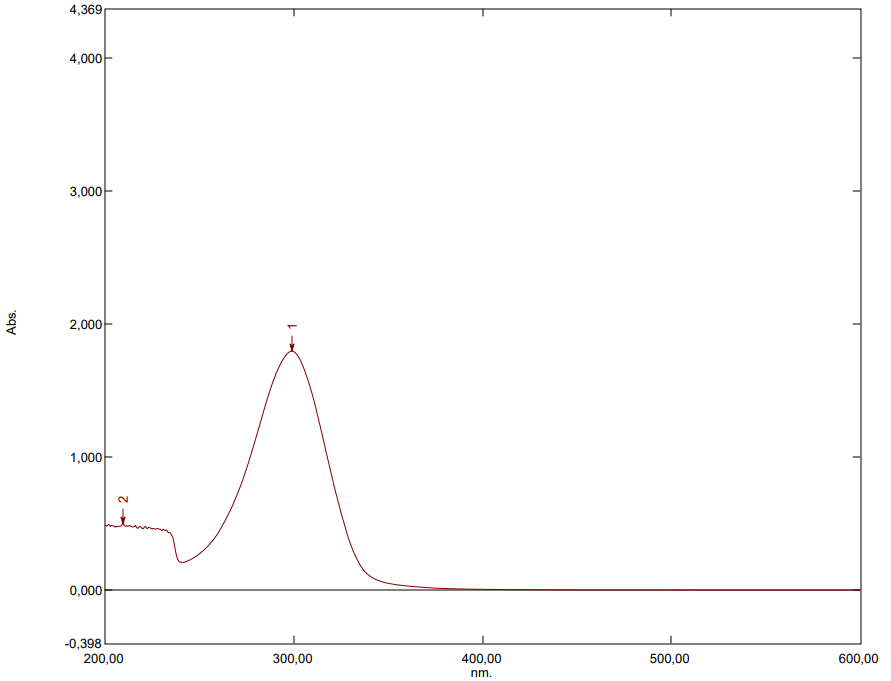


**Figure S19. UV spectra of compound 2 dissolved in CHCl_3_ at the concentration of 0.05 mg/mL.**


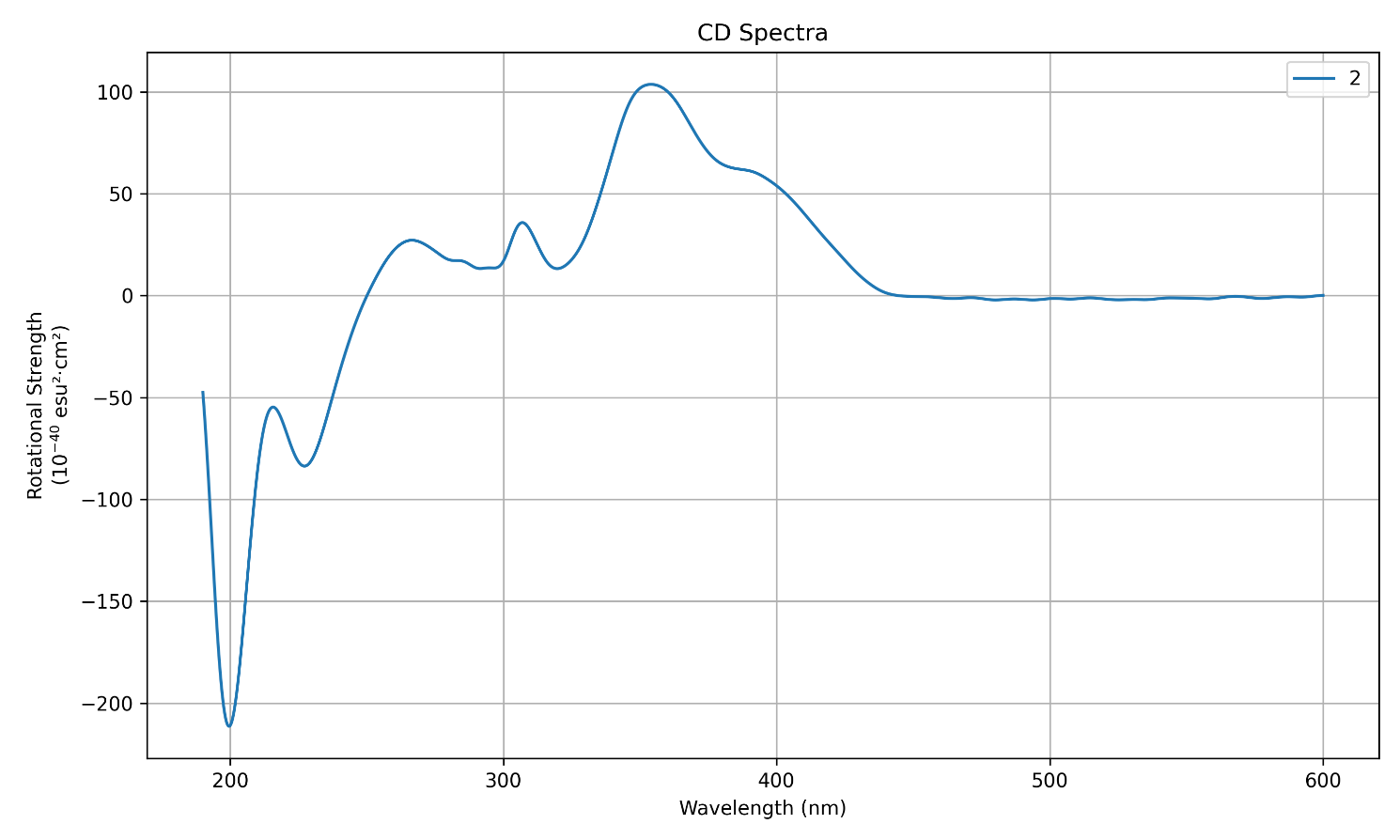


**Figure S20. ECD spectrum of compound 2.**

**Figure S21. ^1^H NMR spectrum of compound 2 (500 MHz) in CDCl_3_.**

**Figure S22. ^13^C NMR Spectrum for compound 2 (125 MHz) in CDCl_3_.**

**Figure S23. ^1^H-^1^H COSY spectrum for compound 2 (500MHz) in CDCl_3_.**

**Figure S24. ^1^H-^13^C HMBC spectrum for compound 2 (500 MHz) in CDCl_3_.**

**Figure S25. HSQC spectrum for compound 2 (500 MHz) in CDCl_3_.**

**Figure S26. ^1^H-^1^H NOESY spectrum for compound 2 (500 MHz) in CDCl_3_.**

**Figure S27. HR-ESIMS spectrum of compound 3.**

**Figure S28. ^1^H NMR Spectrum for compound 3 (500 MHz) in CDCl_3_.**

**
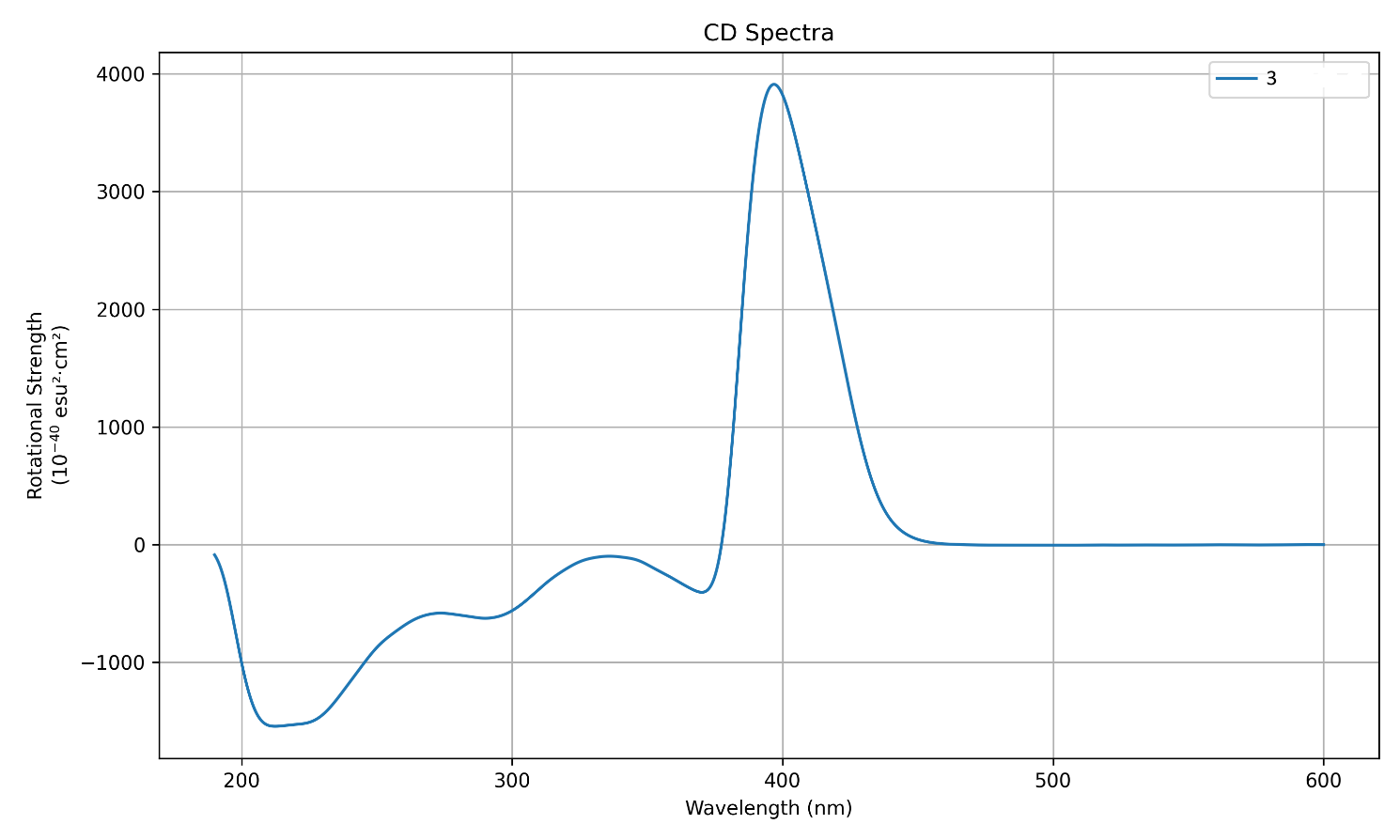
**

**Figure S29. ECD spectrum of compound 3.**

**Figure S30. LR-ESIMS spectrum of compound 4.**

**Figure S31. HR-ESIMS spectrum of compound 4.**

**Figure S32. ^1^H NMR Spectrum for compound 4 (500 MHz) in CD_3_CN.**


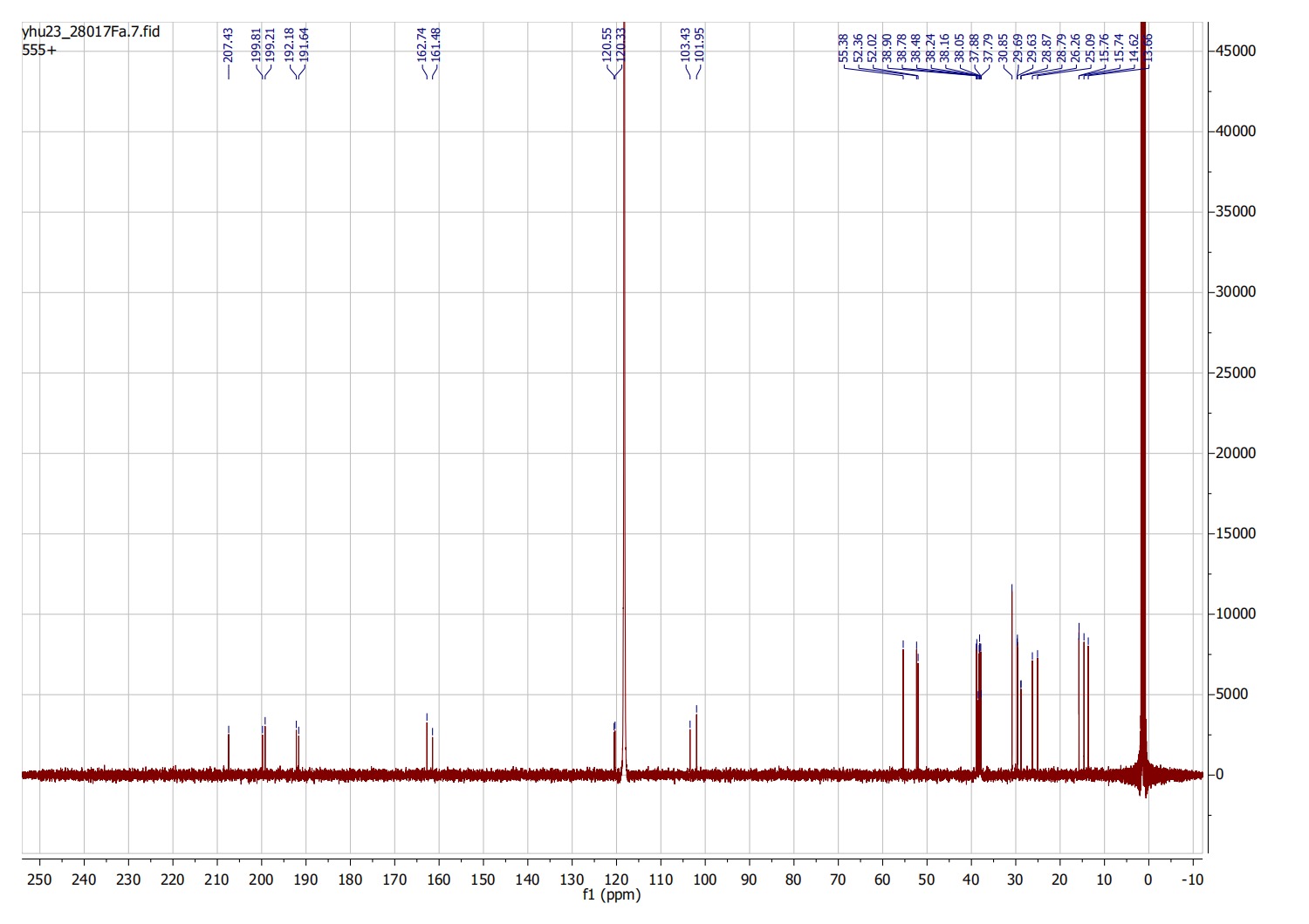


**Figure S33. ^13^C NMR Spectrum for compound 4 (125 MHz) in CD_3_CN.**

**
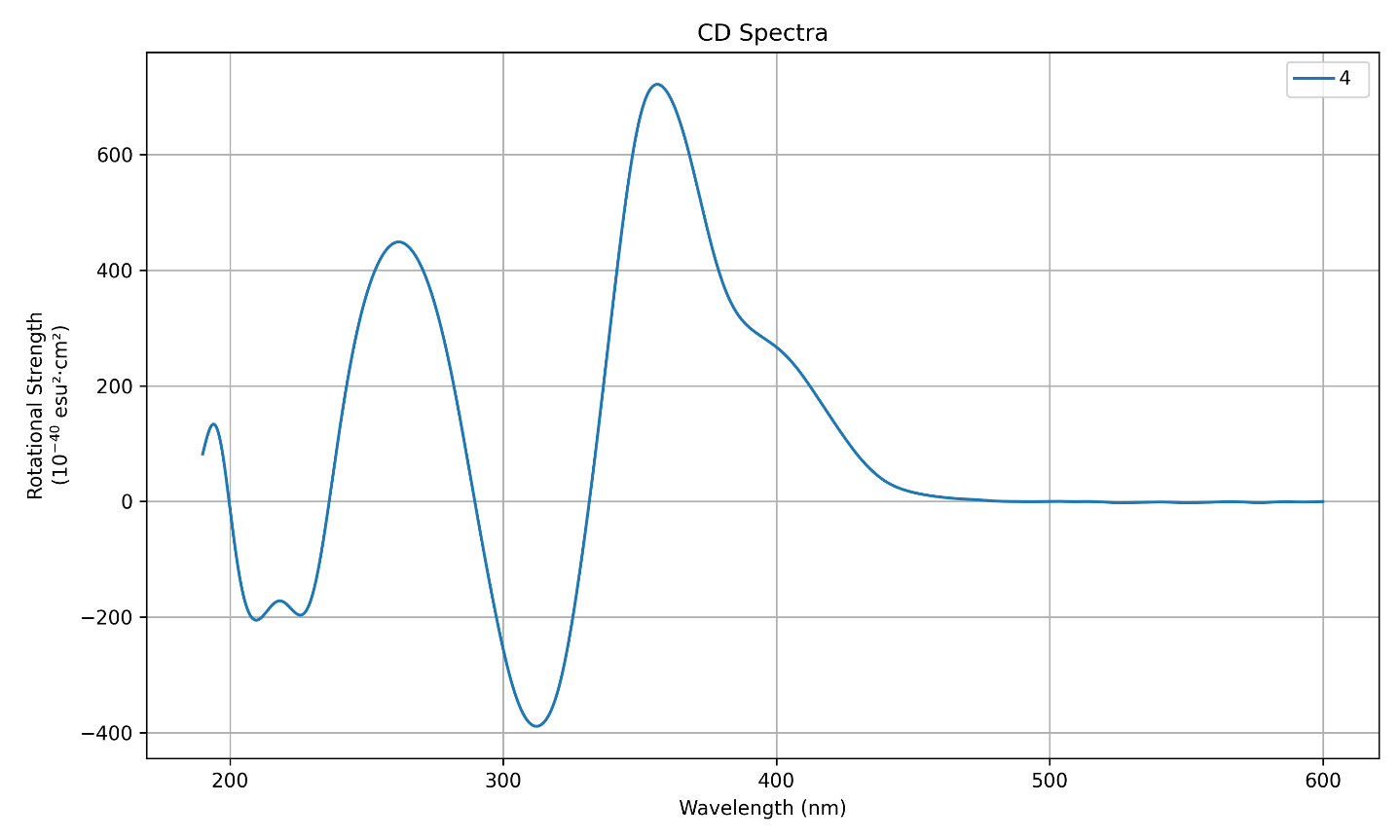
**

**Figure S34. ECD spectrum of compound 4.**


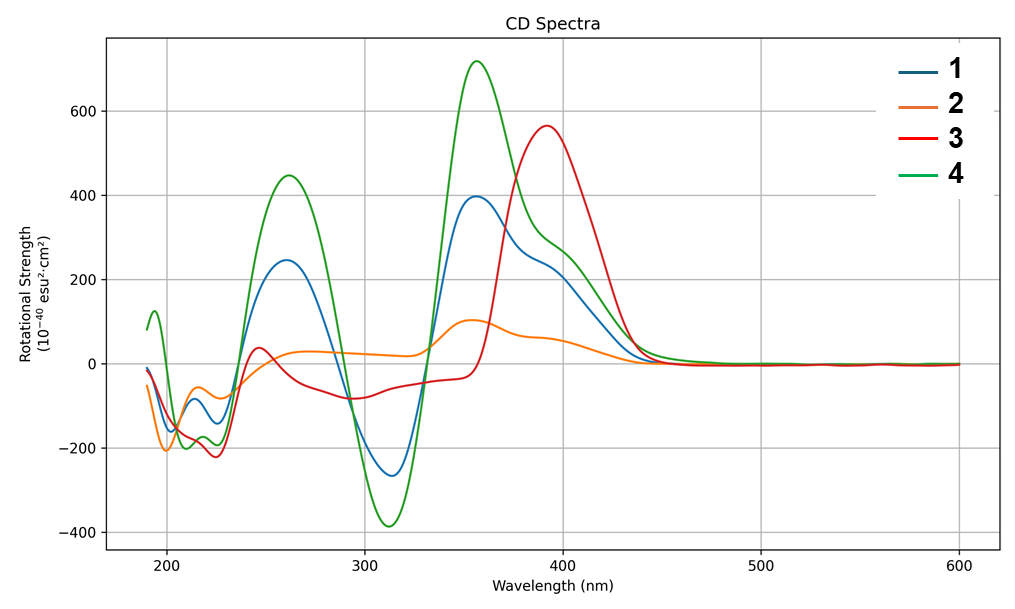


**Figure S35. ECD spectra of compounds 1-4.**

**Figure S36. HR-ESIMS spectrum of compound 5.**

**
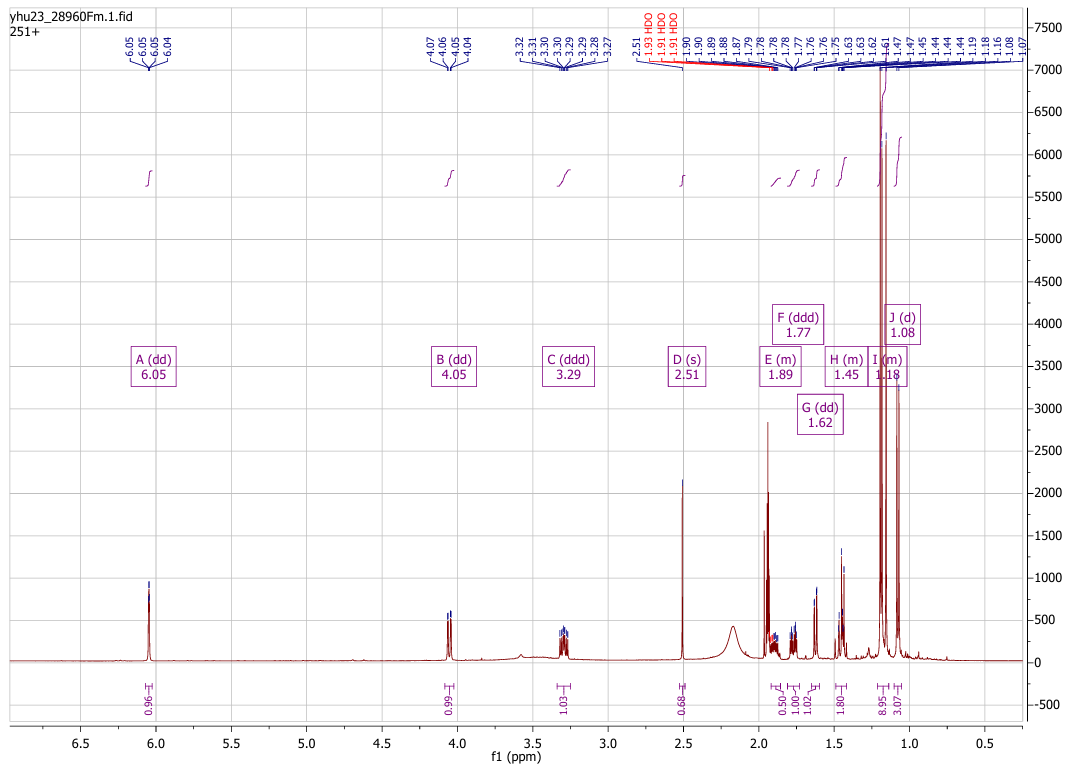
**

**Figure S37. ^1^H NMR Spectrum for compound 5 (500 MHz) in CD_3_CN.**

**Figure S38. HR-ESIMS spectrum of compound 6.**

**Figure S39. ^1^H NMR Spectrum for compound 6 (500 MHz) in CD_3_CN.**

**Figure S40. HR-ESIMS spectrum of compound 7.**

**Figure S41. ^1^H NMR Spectrum for compound 7 (500 MHz) in MeOD.**
